# GLUT1-mediated selective tumor targeting with fluorine containing platinum(II) glycoconjugates

**DOI:** 10.18632/oncotarget.17073

**Published:** 2017-04-13

**Authors:** Ran Liu, Zheng Fu, Meng Zhao, Xiangqian Gao, Hong Li, Qian Mi, Pengxing Liu, Jinna Yang, Zhi Yao, Qingzhi Gao

**Affiliations:** ^1^ Tianjin Key Laboratory for Modern Drug Delivery & High-Efficiency, Collaborative Innovation Center of Chemical Science and Engineering, School of Pharmaceutical Science and Technology, Tianjin University, Tianjin 300072, P. R. China; ^2^ Department of Immunology, Laboratory of Immune Micro-environment, Tianjin Medical University, Tianjin 300070, P. R. China; ^3^ Department of Clinical Laboratory, Tianjin Medical University Cancer Institute and Hospital, National Clinical Research Center for Cancer, Key Laboratory of Cancer Prevention and Therapy, Tianjin's Clinical Research Center for Cancer, Tianjin 300040, P. R. China; ^4^ Affiliated Hospital, Logistics University of the Chinese People's Armed Police Force, Tianjin 300162, P. R. China; ^5^ Department of Medicinal Chemistry, Gudui BioPharma Technology Inc., Huayuan Industrial Park, Tianjin 300384, P. R. China

**Keywords:** fluorine containing platinum(II) glycoconjugates, glucose transporter 1, Warburg effect, tumor targeting

## Abstract

Increased glycolysis and overexpression of glucose transporters (GLUTs) are physiological characteristics of human malignancies. Based on the so-called Warburg effect, ^18^flurodeoxyglucose-positron emission tomography (FDG-PET) has successfully developed as clinical modality for the diagnosis and staging of many cancers. To leverage this glucose transporter mediated metabolic disparity between normal and malignant cells, in the current report, we focus on the fluorine substituted series of glucose, mannose and galactose-conjugated (trans-*R,R*-cyclohexane-1,2-diamine)-2-flouromalonato-platinum(II) complexes for a comprehensive evaluation on their selective tumor targeting. Besides highly improved water solubility, these sugar-conjugates presented improved cytotoxicity than oxaliplatin in glucose tranporters (GLUTs) overexpressing cancer cell lines and exhibited no cross-resistance to cisplatin. For the highly water soluble glucose-conjugated complex (5a), two novel *in vivo* assessments were conducted and the results revealed that 5a was more efficacious at a lower equitoxic dose (70% MTD) than oxaliplatin (100% MTD) in HT29 xenograft model, and it was significantly more potent than oxaliplatin in leukemia-bearing DBA/2 mice as well even at equimolar dose levels (18% vs 90% MTD). GLUT inhibitor mediated cell viability analysis, GLUT1 knockdown cell line-based cytotoxicity evaluation, and platinum accumulation study demonstrated that the cellular uptake of the sugar-conjugates was regulated by GLUT1. The higher intrinsic DNA reactivity of the sugar-conjugates was confirmed by kinetic study of platinum(II)-guanosine adduct formation. The mechanistic origin of the antitumor effect of the fluorine complexes was found to be forming the bifunctional Pt-guanine-guanine (Pt-GG) intrastrand cross-links with DNA. The results provide a rationale for Warburg effect targeted anticancer drug design.

## INTRODUCTION

Since Rosenberg's discovery of cisplatin, [cis-(NH_3_)_2_PtCl_2_] (also known as cis-DDP and CDDP), it has become one of the most potent metal-containing drugs widely used for cancer chemotherapy [1, 2]. Clinical use of cisplatin helps many patients with different types of cancer, such as ovarian cancer, testicular cancer, head and neck tumors, achieve better prognosis and enhanced life quality [3]. However, its poor water solubility and severe side effects including nephrotoxicity, neurotoxicity, ototoxicity, and emetogenesis greatly limit the clinical application [4]. Therefore, improvements to increase solubility and reduce the toxic side effects have drawn extensive research efforts. Compared with cisplatin, the second generation platinum antitumor drug, carboplatin [cis-(NH_3_)_2_Pt(CBDC)] (CBDC, 1,1′-cyclobutyldicarboxylate), reduces the toxic side effects while retaining a similar spectrum of antitumor activity and high extent of cross-resistance with cisplatin [5]. Oxaliplatin, a third-generation platinum analog, has shown a wide range of antitumor activity in metastatic cancer, and shown very good curative effect in the treatment of colorectal cancer [6].

**Figure 1:**
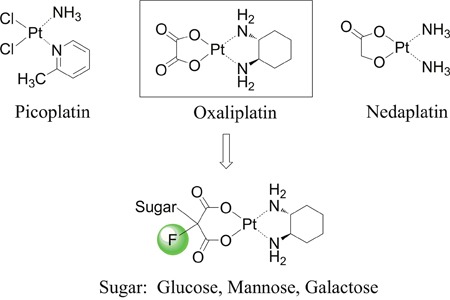
Chemical structures of picoplatin, nedaplatin and platinum(II) complexes in this study

Besides the aforementioned platinum based antitumor drugs, thousands of platinum compounds have been synthesized, and evaluated for their antitumor activity. Various pioneering drug design strategies are deployed to synthesize new antitumor platinum derivatives [7, 8]. These strategies are mainly focused on changing the chelating ligand or altering the leaving groups of the platinum(II) complexes. The chelating ligand, also known as neutral ligand, could substantially avoid resistance, and influence the efficacy as well as the mechanism of cellular uptake of platinum(II) derivatives [9]. For example, picoplatin (Figure 1) [10] has been reported to show low reactivity with glutathione and improved responses in resistant tumors [11, 12].

Furthermore, structural modification at the leaving group also modifies the physicochemical properties (e.g. water solubility and stability) of the complex and is therefore a promising strategy in drug design [13]. Nedaplatin (Figure 1), containing a glycolate structure as the leaving group, exhibits improved outcomes over cisplatin when used in combination with docetaxel in a phase III trial of advanced squamous cell carcinoma (SCC) of the lung [14]. While most of the molecular modification and structural design aim at improving therapeutic efficacy and/or decreasing the side effects, few studies strategically focus on enhancing tumor selectivity.

Compared to non-transformed tissue, cancerous tissues exhibit increased glucose consumption and display high rates of aerobic glycolysis [15, 16]. This glycolysis enhancement phenomenon is known as the Warburg effect. One of the most well-known examples of targeting the Warburg effect is the 2-deoxy-2-(^18^F) fluoro-D-glucose (^18^F-FDG)-based positron emission tomography (PET) imaging technique, which has been widely and successfully used in cancer diagnosis and clinical staging [17, 18].

Biological studies demonstrated that abnormal or impaired glucose homeostasis is not only involved in diabetes [19, 20], but also in cancers. To achieve increased glucose consumption, the insulin-independent glucose transporter GLUT1, is widely overexpressed in the majority of human cancers [21, 22], which makes them attractive therapeutic targets. With the aim of developing a new class of selective tumor-targeting platinum(II) anticancer agent, our principal interest is to leverage the GLUT1 mediated selective drug delivery as an anticancer strategy. Therefore, our design concept is to increase transportability and intra-tumoral accumulation of the platinum(II) complexes through glycoconjugation at the malonato leaving ligand which may help the drug molecule be recognized by GLUTs (Figure 1). At the same time, introduce of sugar moiety and fluorine substitution will largely enhance water solubility of the platinum(II) complexes and diminish non-selective drug uptake.

GLUT1 is a facilitative sugar transporter and primarily transport glucose. Galactose, mannose as well as glucosamine can also be transported by GLUT1 with distinct efficiencies [23]. Through our previous proof-of-concept studies, we established some useful synthetic and basic biological evaluation methods on different platinum-glycoconjugates including C1-O-glycosides [24], 2-deoxy and 6-deoxy-glycoconjugates [25, 26], as well as developed fluorescent bioprobes as useful tool for cell-based glucose uptake imaging [27].

In 2013, we reported the first sugar conjugated oxaliplatin type platinum(II) glycoconjugates with high water solubility and antitumor activity comparable to non-sugar conjugated oxaliplatin. We found that the fluorine substitution has a profound effect on water solubility improvement of the platinum(II) complex [28]. In the present article, we report the comprehensive biological, pharmacological and mechanistic studies on a series of 2-flouromalonato-platinum(II) glycoconjugates using glucose, mannose and galactose as GLUT targeting sugar motif. For a comparable reason, the same chelating ligand DACH as of oxaliplatin was adopted in the structure and all evaluations were performed along with assessments of oxaliplatin. All complexes were investigated for water solubility measurement and cytotoxicity analysis in six different cancer cell lines. The study also revealed, for the first time, the glucose conjugated platinum(II) complex 5a exhibits superior *in vivo* efficacy to oxaliplatin at equimolar dose level (as low as 18% of the MTD for 5a vs. 90% MTD for oxaliplatin) in mouse ascetic leukemia model, and at a lower equitoxic dose level in another colon cancer xenograft model. In order to gain a deeper insight into the mechanism of action, GLUT mediated cell uptake, DNA interaction and DNA binding kinetics as Warburg effect targeting properties of the new compounds were investigated.

## RESULTS

### Chemistry

**Figure 2:**
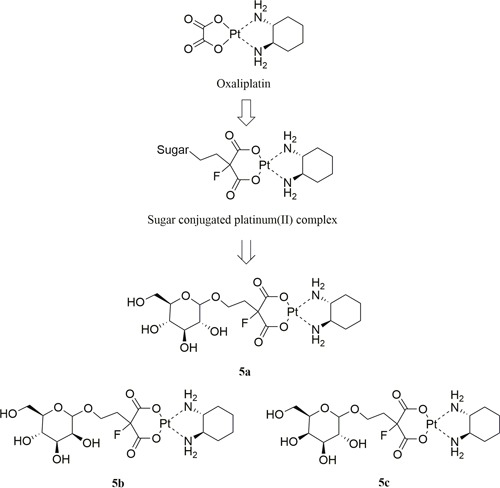
Design concept and chemical structure of sugar conjugated platinum(II) complexes Glucose-conjugated (trans-*R,R*-cyclohexane-1,2-diamine)-2-flouromalonato-platinum(II) complex (**5a**); Mannose-conjugated (trans-*R,R*-cyclohexane-1,2-diamine)-2-flouromalonato-platinum(II) complex (**5b**); Galactose-conjugated (trans-*R,R*-cyclohexane- 1,2-diamine)-2-flouromalonato-platinum(II) complex (**5c**).

**Scheme 1:**
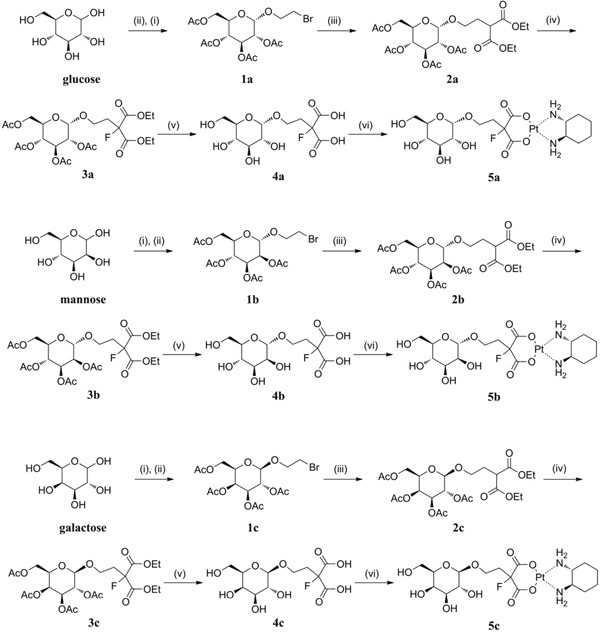
Synthesis of sugar conjugated platinum(II) complexes^a^ ^a^Reagents and conditions: (i) Ac_2_O, Py.; (ii) ethylene bromohydrin, BF_3_·Et_2_O; (iii) diethylmalonate, K_2_CO_3_, DMF; (iv) Selectfluor, NaH, DMF; (v) NaOH, MeOH/H_2_O, 90°C; (vi) Ba(OH)_2_·8H_2_O, Pt(DACH)SO_4_, H_2_O, 25°C. Compound **5a** was previously reported [31].

Three sugar-conjugated (trans-*R,R*-cyclohexane- 1,2-diamine)-2-flouromalonato-platinum(II) complexes (5a, 5b, 5c) have been synthesized from glucose, mannose and galactose respectively (Figure 2). The structures of all three complexes were confirmed by NMR (^1^H and ^13^C) spectroscopy, infrared spectroscopy and high resolution mass spectra (HRMS, m/z). The schematic presentation of the reaction for the syntheses is shown in Scheme 1. The synthesis of the glucose-conjugated (trans-*R,R*-cyclohexane- 1,2-diamine)-2-flouromalonato-platinum(II) complex (5a), mannose-conjugated (trans-*R,R*-cyclohexane- 1,2-diamine)-2-flouromalonato-platinum(II) complex (5b), and galactose-conjugated (trans-*R,R*-cyclohexane- 1,2-diamine)-2-flouromalonato-platinum(II) complex (5c), was accomplished in a five-step sequence that started with D-glucose, D-mannose and D-galactose respectively. The corresponding 2-bromoethyl-2,3,4,6-tetraacetyl-D-glycoside were prepared according to the literature method [29, 30], followed by electrophilic substitution reaction with diethylmalonate through a two carbon linkers. The introduction of fluorine to the 2-position of malonic acid was performed using selectfluor as fluorinating reagent, which could inhibit the enolization of malonic acid, to increase stability of the malonatoplatinum complexes and improve the water solubility on account of fluorine mediated hydrogen bonding interaction. The sugar conjugated malonic acid ligand was obtained though hydrolyzation and purified by preparative HPLC in high yields.

The coupling reactions of sugar conjugated malonic acid ligand with (trans-*R,R*-cyclohexane-1,2-diamine) sulfatoplatinum(II) afforded previously described compound 5a [31] and mannose-conjugated platinum(II) complex 5b and galactose-conjugated platinum(II) complex 5c in moderate to high yields. All final platinum(II) complexes 5a, 5b and 5c were obtained as white solid with a total yield of 58.3%, 70.2% and 69% for the final step respectively and the purity can reach up to 98.46% after preparative HPLC purification.

**Figure 3:**
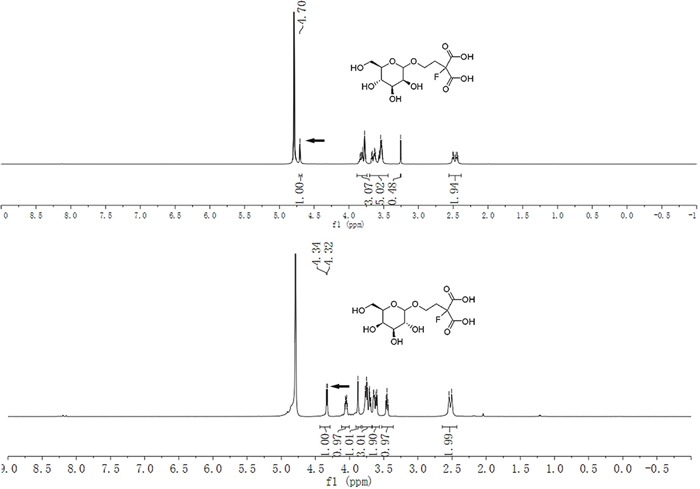
^1^H NMR spectra of the anomeric configuration of the mannose and galactose conjugated malonic acid ligands

The stereochemistry for compound 5b and 5c were determined at the stage of sugar conjugated malonic acid ligands. As illustrated in Figure 3, ^1^H NMR analysis with the proton on 1-position of the sugar moiety, and the mannose derived glycoside was assigned as a pure α-anomer (4b: δ 4.70 (s, 1H), 600 MHz, D_2_O) [30] whereas the galactose derived glycoside was assigned as a pure β-anomer (4c: δ 4.33 (d, *J* = 7.8 Hz, 1H), 600 MHz, D_2_O)

### Water solubility

Poor water solubility and low lipophilicity of platinum mediated anti-tumor drugs have brought great inconvenience in clinical application. In the current study, by introducing the highly polar sugar moiety and the fluorine as a hydrogen bonding acceptor (which helps the slvation process) to the structure of platinum complexes, the water solubility of the drug molecule was considered can be significantly improved.

The water solubility of sugar conjugated platinum complexes was determinated and compared to that of oxaliplatin and cisplatin. As summarized in Table 1, glycosylation of the platinum complexes dramatically improved the water solubility. Solubility of 5a achieved 946.8 mg/mL, 155 fold higher than oxaliplatin, 946 fold higher than cisplatin. The relatively insignificant increase was observed for 5c, but it still exhibited 108 times increase compared with oxaliplatin. As compared with methyl containing platinum(II) glycoconjugates that we described in our previous research [32], the fluorine containing platinum(II) glycoconjugates exhibited even better water solubility. The most obvious enhancement could be observed from 5b, whose solubility was improved almost 16 times over the methyl substituted complex: (trans-*R,R*-cyclohexane-1,2-diamine)-2-methylmalonatoplatinum(II)-α-D-mannose conjugate (Man-Me-Pt).

**Table 1:** Water solubilities of fluorine containing platinum(II) glycoconjugates at 25°C

Complex	Molecular Weight	Solubility (mg/mL)^a^	Complex	Solubility (mg/mL)^b^	Ratio-Me ^c^	Ratio-Oxa ^d^	Ratio-Cis ^e^
**5a**	635.5	946.8	Glu-Me-Pt	531.0	1.8	155.2	946.8
**5b**	635.5	868.2	Man-Me-Pt	54.5	15.9	142.3	868.2
**5c**	635.5	661.3	Gal-Me-Pt	279.5	2.4	108.4	661.3
Oxaliplatin	397.3	6.1				1.0	
Cisplatin	300.0	1.0					1.0

^a^ water solubility of complexes presented in column 1.

^b^ water solubility of complexes presented in column 3.

^c^ water solubility ratio of fluorine containing platinum(II) glycoconjugates versus methyl containing platinum(II) glycoconjugates which described in our previous research.

^d^ water solubility ratio of fluorine containing platinum(II) glycoconjugates versus oxaliplatin.

^e^ water solubility ratio of fluorine containing platinum(II) glycoconjugates versus cisplatin.

### *In vitro* cytotoxicity

To identify the *in vitro* anti-cancer activity of compounds 5a, 5b and 5c, the cytotoxicity of these complexes in comparison to oxaliplatin was assessed by means of MTS assay in six human carcinoma cell lines. The corresponding IC_50_ values are given in Table 2. Each complex showed varying cytotoxic profiles. In general, the range of *in vitro* cytotoxicity determined for the platinum sugar conjugates was comparable to oxaliplatin. However, there is not a correlation between hydrophilic property of platinum complexes and their cytotoxic effects. 5a exhibited 2-fold higher activity than oxaliplatin in A549 human lung cancer cell line. In SK-OV-3 epithelial ovarian cancer cell line, 5b showed higher anti-tumor efficacy than oxaliplatin, as reflected by up to 70% lower IC_50_ values. All three compounds presented effective anti-tumor activity in lung cancer (H460), as shown by their IC_50_ values, which are essentially half of oxaliplatin. We observed similar results in breast cancer (MCF7), with the exception of 5c complex, where oxaliplatin was more active. Although all cell lines responded to all anti-proliferative complexes, the colon cancer cell line HT29 was the most sensitive. As depicted in Table 2, the cytotoxicity of 5a is almost 10-fold higher in HT29 cells than oxaliplatin, whereas the cytotoxic effect of 5c and 5b was about 5-fold and 2-fold higher respectively. In addition, to mitigating undesired toxic side effects associated with chemotherapy, we evaluated the selectivity of 5a and oxaliplatin using EBAS-2B-CM (human lung epithelial cells). EBAS-2B-CM cells were exposed to 6.25 μM of oxaliplatin and platinum complex 5a for 72 h. MTS assay was then conducted to determine the percentage of the living cells. The results showed that compared with oxaliplatin, compound 5a didn't exhibited cytotoxicity against EBAS-2B-CM cells, indicating lower toxic effects and better selectivity of 5a betwwen normal and cancer cells (Supplementary Figure 30).

**Table 2:** Cytotoxicity of fluorine containing platinum(II) complexes given as mean ± SD in six human cancer cell lines in comparison with Oxaliplatin*

Compounds	IC_50_ (μM)					
	HT29	H460	DU145	A549	SKOV3	MCF7
Oxaliplatin	5.05 ± 0.20	22.11 ± 0.17	2.05 ± 0.16	0.86 ± 0.09	8.54 ± 0.18	0.79 ± 0.11
**5a**	0.53 ± 0.16	11.05 ± 0.11	5.98 ± 0.16	0.35 ± 0.17	16.66 ± 0.32	0.61 ± 0.13
**5b**	2.16 ± 0.18	10.21 ± 0.15	2.60 ± 0.18	0.65 ± 0.10	2.22 ± 0.19	0.41 ± 0.09
**5c**	1.07 ± 0.12	10.04 ± 0.15	5.11 ± 0.14	4.90 ± 0.16	22.05 ± 0.20	9.82 ± 0.12

*The IC_50_ value for Cisplatin in HT29 cells was 4.05 ± 0.11 μM. IC_50_ was evaluated by the MTS assay for 72 h. All compounds were tested in the same batch and the experiments were performed three times with five replicates. HT29 (human colon cancer cell line), H460 (human lung cancer cell line), DU145 (human prostate cancer cell line), A549 (human lung carcinoma), SKOV3 (human ovarian cancer cell line), MCF7 (human breast cancer cell line).

**Figure 4:**
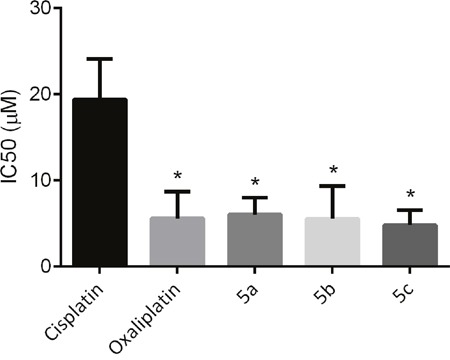
IC_50_ of cisplatin, oxaliplatin, 5a, 5b and 5c against cisplatin resistant HT29cisR human colon carcinoma cell line (* p < 0.05 compared to cisplatin group)

The mechanisms of cellular cisplatin resistance which have been investigated to date include the following components: decreased drug accumulation, increased drug inactivation, enhanced DNA repair/tolerance, and conjugation to intracellular thiols like glutathione (GSH) [33]. Sugar conjugated platinum(II) complexes can cross cell membrane facilitated by GLUT1 mediated active transport (see section Cellular uptake mechanism of the platinum complexes), the leaving ligand (sugar-derived malonate) will hydrolyzed inside the cell, therefore forming aquocomplexes and affording the same active principle as oxaliplatin. On the basis of this hypothesis, we expect that the current platinum(II) glycoconjugates will be able to circumvent cross-resistance to cisplatin. The cytotoxic activities of oxaliplatin and sugar conjugates were determined in both the cisplatin resistant HT29cisR cell line and the cisplatin sensitive HT29 human colon carcinoma cell line. As shown in Figure 4, unlike cisplatin, the sugar conjugated platinum(II) complexes displayed high cytotoxicity against human colon HT29cisR cell line, with IC_50_ values in the range of 4.78-6.02 μM in contrast to 19.35 μM of cisplatin.

### Cellular uptake mechanism of the platinum complexes

**Figure 5:**
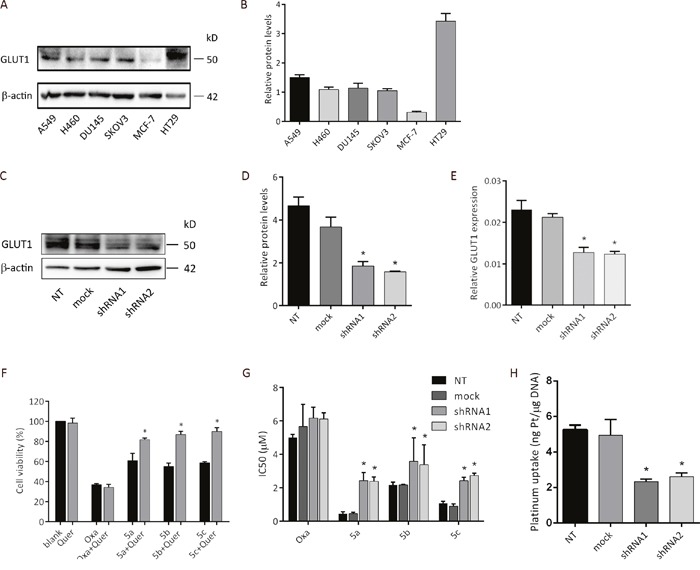
Cellular uptake mechanism of the platinum complexes **(A)** Expression of GLUT1 in various cancer cell lines determined by western blot analysis. **(B)** Bar graph for the quantitative comparison among GLUT1 expression levels. **(C)** GLUT1 expression levels in empty vector transfected and shGLUT1-transfected cells confirmed by western blot. **(D)** Bar graph for the quantitative comparison among GLUT1 expression levels in empty vector transfected and shGLUT1-transfected cells. **(E)** RT-PCR analysis of GLUT1 mRNA expression in empty vector transfected and shGLUT1-transfected cells. NT: HT29 cells; mock: HT29 cells transfected by empty vector pLKO.1; shRNA1: HT29 cells transfected by shGLUT1-1 and selected by 1 μg/mL of puromycin; shRNA2: HT29 cells transfected by shGLUT1-2 and selected by 1 μg/mL of puromycin. **(F)** Cytotoxicity of oxaliplatin and glucose conjugated platinum complexes against HT29 human colon cancer cell line in the presence and in the absence of GLUT1 inhibitor quercetin. MTS assay showing cell viability of HT29 after treated with oxaliplatin and glucose conjugated platinum complexes (100 μM) alone or together with quercetin (20 μM) for 48h. HT29 exposed with PBS or quercetin alone were served as negative control. **(G)** HT29, HT29-mock and HT29 transfected by shGLUT1 cells were treated with oxaliplatin and three sugar-conjugated platinum complexes for 72h, and the IC_50_ values were determined by the MTS assay. **(H)** Platinum uptake after 2 h treatment of compound **5a** at 200 μM. Data was calculated from three independent experiments. *P < 0.05, compared to the control group.

Many small molecule drugs can diffuse across the lipid bilayer easily. However, larger and more hydrophilic molecules cannot pass through the cell membrane by simple diffusion. These substances can just be transported by special proteins [34]. Based on this theory and our design concept, we compared differential GLUT1 protein expression six cancer cell lines and postulated that the improved cytotoxicity of water-soluble sugar conjugate 5a was due to the active uptake by GLUT1. GLUT1 was analyzed by western blotting of whole-cell extracts. Figure 5A revealed that human colon cancer cell line HT29 had the highest expression of GLUT1. Interestingly, the expression levels correlated with the anti-proliferation activity against HT29 for all three platinum complexes, indicating that the transportation of platinum complexes might be mediated by GLUT1.

To test the hypothesis, we performed the inhibitor dependent cytotoxicity assay of sugar conjugate 5a and oxaliplatin by using quercetin as the inhibitor. Human colon cancer cell line HT29 was chosen for this study as it showed the highest expression level of GLUT1 protein among all tested cell lines. HT29 cells were grown in monolayer and were treated with and without quercetin, followed by exposure to 100 μM of platinum(II) complexes for 48 h. MTS assay was used to analyze the cell viability and the effects of GLUT1 inhibitor quercetin on cytotoxicity. As depicted in Figure 5D, the cell-killing potency of all three sugar conjugates revealed significant dependency on quercetin (* P < 0.05). However, under the same condition, quercetin alone did not exert any cytotoxic effect.

Similar improvement of the anti-proliferation activity of platinum complexes was also observed when GLUT1 expression was down-regulated by short-hairpin RNAs (shRNA). HT29 cells transfected by empty vector pLKO.1 (HT29-mock) was used as control. Retroviral-mediated gene transduction of shRNA was used to generate GLUT1 knockdown clones (HT29-shRNA). To examine GLUT1 knock-down efficiency, RT-qPCR and western blot analyses were performed (Figure 5C, 5D and 5E). In consistence with the inhibitory effect of quercetin, the IC_50_ values of all three sugar conjugates were strikingly increased in HT29-shRNA cells, as depicted in Figure 5E. However, the antitumor efficacy of oxaliplatin showed no significant difference between HT29-mock and HT29-shRNA cells.

Cellular platinum accumulation analysis of compound 5a was tested in HT29, empty vector transfected and shGLUT1-transfected cells. After incubation for 2 hours at 200 μM of 5a, DNA was isolated and the intracellular platinum accumulation was measured by inductively coupled plasma mass spectrometry (ICP-MS). The uptake values are depicted in Figure 5H. Interestingly, the uptake of compound 5a in HT29 and HT29-mock cells was greater than that in HT29-shRNA1 and HT29-shRNA2 cells, which is also consistent with the observed cytotoxic activity.

### Intrinsic DNA reactivity of the sugar conjugates

To further understand the intrinsic reactivity of our synthesized sugar conjugated platinum complexes with DNA, the kinetic study of platinum complexes with 5′-Guanosine Monophosphate (5′-GMP) implementing ^1^H NMR analysis was performed to mimic the platinum(II)-DNA adduct formation. 5′-GMP can coordinate to metal ions through N1 and N7 atoms, but only bind through the N7 position at pH 7.2 cause the N1 atom is protonated (pKa (N1) = 9.3) in this medium [35]. For our sample system, in which the pH had been adjusted to 7.3, the coordination of 5′-GMP to platinum occurred via N7 position. The reaction of platinum complexes with 5′ -GMP can be monitored by the typical downfield shift of H8 proton on 5′-GMP from its free form in ^1^H NMR spectrum.

**Figure 6:**
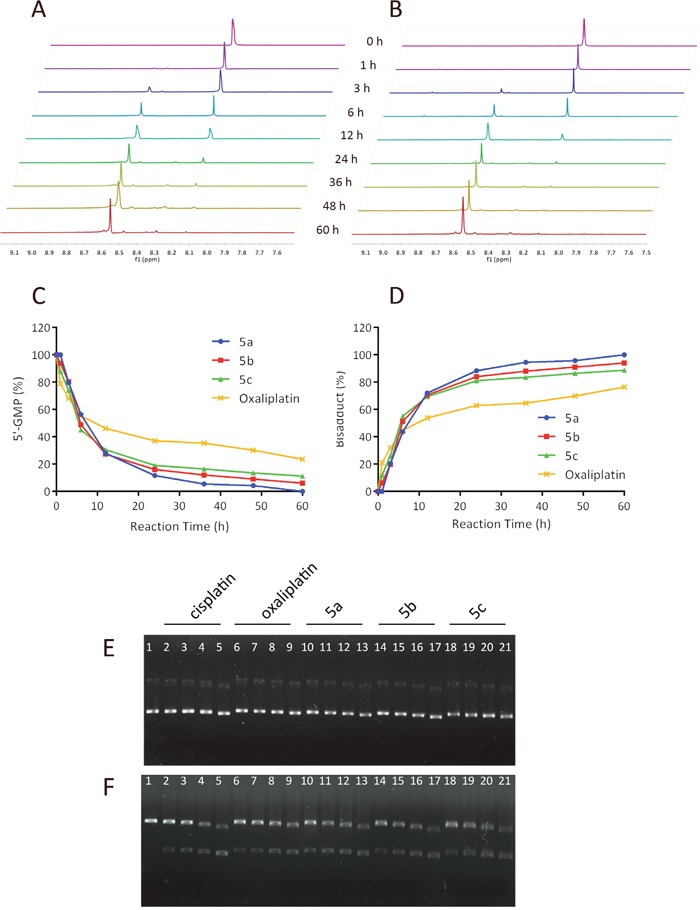
Intrinsic DNA reactivity of the sugar conjugates and DNA modification mechanism analysis Kinetics of the reaction of 5′-GMP with oxaliplatin **(A)** or **5a (B)** in a molar ratio of 2:1 in deuterium oxide at 37°C investigated using ^1^H NMR. Time course of 5′-GMP consumption **(C)** and bisadducts formation **(D)** Electrophoretograms of pUC18 plasmid DNA incubated with varying concentrations of cisplatin, oxaliplatin and sugar conjugated platinum complexes **5a**, **5b**, **5c** ranging from 12.5 to 100 μM, followed by BamHI digestion. **(E)** Lane 1 applies to the untreated pUC18 plasmid DNA, lane 2-5: apply to pUC18 plasmid DNA interacted with increasing concentrations of cisplatin (12.5, 25, 50, 100 μM, respectively), lanes 6–9: apply to pUC18 plasmid DNA interacted with increasing concentrations of oxaliplatin (12.5, 25, 50, 100 μM, respectively), lane 10-13: apply to pUC18 plasmid DNA interacted with increasing concentrations of **5a** (12.5, 25, 50, 100 μM, respectively), lane 14-17: apply to pUC18 plasmid DNA interacted with increasing concentrations of **5b** (12.5, 25, 50, 100 μM, respectively), lane 18-21: apply to pUC18 plasmid DNA interacted with increasing concentrations of **5c** (12.5, 25, 50, 100 μM, respectively). **(F)** BamHI digestion of the incubated mixtures of pUC18 plasmid DNA as indicated in Figure A.

The reactivity of platinum complexes as well as oxaliplatin (5 mM) towards 5′-GMP (10 mM) was investigated with a pH of 7.2-7.4 at 37°C. A 5 mM solution of the sodium chloride was added to the mixture to simulate the intracellular environment. As summarized in Figure 6C and 6D, 5a, 5b and 5c exhibited faster reactions with nucleophlic base 5′-GMP compared to oxaliplatin.

### DNA modification mechanism analysis

N7 of guanine (G) is the most electron-rich site among all the DNA's nucleophilic centers, which the active [Pt(L)2]^2+^ species may bind to and form DNA cross-linkage [13]. To assess if the *in vitro* anti-proliferative activity of the sugar conjugates may be at the DNA adduct level, a plasmid unwinding assay was performed. The local untwisting of closed-circular supercoiled DNA induced by different types of platinum−DNA adducts can be determined by this assay [36]. In this experiment, gel electrophoresis was applied to analyze the interaction between pUC18 plasmid DNA and platinum complexes for 12 h at 37°C of pUC18 plasmid DNA with increasing concentrations of 5a, 5b, 5c, cisplatin and oxaliplatin ranging from 12.5 to 100 μM. pUC18 plasmid DNA was initially found to be a mixture consists of supercoiled form I (the major form) and singly-nicked form II (the minor form). On agarose gels, supercoiled plasmid DNA (form I) migrates faster in an electrical field than the nicked circle (form II). When pUC18 plasmid DNA was treated with sugar-conjugates, cisplatin and oxaliplatin, the mobility of supercoiled forms and singly-nicked form plasmid DNA bands increased and showed concentration dependency. As illustrated in Figure 6E, the two bands of forms I and II almost moved at the same rate in all tested compounds and remained essentially parallel at all concentrations. In addition, the moving distance of the two bands increased as the concentration increased, exhibiting concentration dependency. There exist no significant differences between these platinum complexes which behaved large in common with cisplatin.

The change in mobility of plasmid DNA bands caused by interaction with cisplatin is due to the platination of the bases by intrastrand bifunctional GG binding. pUC18 plasmid DNA could be digested with restriction enzyme BamHI at the specific GG site to construct linear form III DNA [37]. Figure 6F gives the electrophoretograms incubated with mixtures of pUC18 plasmid DNA at various concentrations of sugar conjugates, oxaliplatin and cisplatin, followed by BamHI digestion. For untreated pUC18 plasmid DNA, BamHI digestion only produced the form III DNA. However, BamHI digestion was prevented at all concentrations of the compounds since no form III DNA was observed, suggesting that our compounds formed bifunctional GG binding DNA adducts as cisplatin and oxaliplatin did.

### Maximum tolerated dose (MTD) determination and antileukemic activity of complex 5a *in vivo*

**Figure 7:**
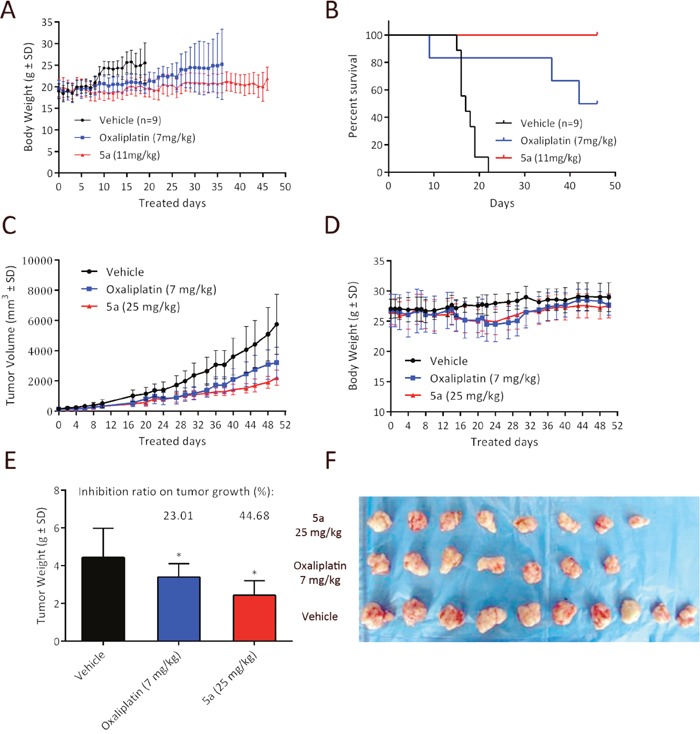
*In vivo* anticancer activity of 5a in ascetic leukemia L1210 bearing DBA/2 mice model and HT29 bearing BALB/c nude mice model **(A)** Body weight curve for **5a** (11 mg/kg) and oxaliplatin (7 mg/kg) in L1210 leukemia bearing DBA/2 mice. **(B)** Kaplan−Meier curve showing the survival curves of L1210 leukemia-bearing mice treated via intra-peritoneal injection (i.p.) with **5a** (11 mg/kg) and oxaliplatin (7 mg/kg) on days 1, 5, 9 and compared to that of normal saline treated controls (n = 9). **(C, D)** Growth inhibition effect of **5a**, oxaliplatin and vehicle in HT29 xenograft model. HT29 tumor xenograft was treated on days 0, 7, 14, and 21 with **5a** (25 mg/kg, 70% MTD), oxaliplatin (7 mg/kg, 100% MTD) and saline via intravenous injection. Tumor volume and body weight curve over time in BALB/c nude mice. **(E)** The tumor weight of each group was measured at the time of sacrifice, and was calculated as % tumor growth inhibition (%TGI). (* p < 0.05 compared to control group) **(F)** Photographs of the autopsied tumors from each treatment group.

Based on *in vitro* cytotoxicity, compound 5a was chosen for further investigations. In the previous study, we conducted the maximum tolerated dose (MTD) experiments in immunodeficient BALB/c nude mice and DBA/2 mice to evaluate their toxicity profiles [24, 31]. The maximum tolerated dose is defined as the highest tolerated dose (mean weight loss < 15%) without causing major (life-threatening) toxicity (< 15% toxic deaths) in the study. Both the MTD values of these two mouse models are nearly 5-fold higher than those of oxaliplatin (DBA/2: 60 mpk for 5a vs. 10 mpk for oxaliplarin; BALB/c: 36 mpk for 5a vs. 7 mpk for oxaliplatin). In our previous work, we conducted a comparison study to check the survival benefit of 5a over oxaliplatin under equitoxic conditions (both for 5a and oxaliplatin at 70% of their MTD dosages). In this work, using L1210 leukemia bearing DBA/2 mice, we challenged to further reduce the dosage of 5a to an equal molar level (11 mpk for 5a as of 18% MTD vs. 7 mpk for oxaliplatin as of 70 % MTD). To the best of our knowledge, this is the first case that the platinum complexes which exhibit significantly different water solubility to be compared on an equal molar basis. As summarized in Figure 7A and 7B, it was found that treatment of equal molar of 5a still recorded overwhelming superiority in lifespan and survival rate.

### *In vivo* growth inhibition of HT29 xenograft model

Since complex 5a being the most potent compound in the series in cytotoxicity against HT29, its *in vivo* anticancer activity was examined in HT29 tumor xenograft BALB/c nude mice model. The dose regimen was set as: 5a, 25 mg/kg at 70% of its MTD vs. oxaliplatin, 7 mg/kg at 100% of its MTD, with the schedule of qw x 4 and monitored for 51 days. As shown in Figure 7C the treatment with 5a at 25 mg/kg resulted in statistically significant tumor shrinkage. The tumor growth inhibition (TGI) of 5a was greater than that of oxaliplatin. With regard to the tumor weight change, similar results were observed (Figure 7E). 5a at a dose of 25 mg/kg caused significant tumor shrinkage and the reduction in tumor weight (TGI, 44.68%, * P < 0.05 vs. control group) was greater than that of oxaliplatin (TGI, 23.01%). Throughout the treatment period, no signs of toxicity such as distress or fatigue were observed and there were no significant difference in body weight change among the vehicle group, 5a and oxaliplatin-treated groups (Figure 7D). In addition, 90% maximal tolerated dose of oxaliplatin was applied in this experiment, whereas 5a was applied at 70% maximal tolerated dose. These observations collectively suggest 5a strongly inhibits tumorigenesis in colon cancer xenografts *in vivo*, even at a relatively low dose.

## DISCUSSION

The rapid growth of cancer cells demands large amounts of glucose and drastic increases in glucose metabolite flux. Today this phenomenon is known as the “Warburg effect” and is one of the hall marks of cancer [16]. The altered glucose metabolism and glucose transporter overexpression are common in cancer cells and provide promising targets for anti-cancer drugs [38]. Oxaliplatin has been widely used in clinical application for the treatment of different types of cancer, such as colon cancer, urothelial cancer, and breast cancer. It can achieve better prognosis and improve life quality [39, 40]. However, it may cause severe side effects including hyperalgesia, motor dysfunctions and peripheral neuropathy [4, 41, 42], which partially originated from a lack of tumor selectivity. With the ever-increasing number of potential targets, it is imperative to choose the right target for drug discovery in the development of target-selective therapeutics [43, 44]. Novel strategies, such as glycoconjugation, for enhancing tumor-targeting properties of platinum anti-cancer complexes are of great interest and become an appealing strategy for targeted delivery [45, 46]. The radiolabeled glucose derivative 18F-FDG is a successful representative of glycoconjugation in tumor diagnosis and is supported by GLUTs-mediated uptake and selective tumor accumulation. However, few glycoconjugated anti-cancer drugs have been synthesized for targeted cancer therapy. In the current study, a series of sugar conjugated platinum complexes with specific tumor-targeting properties were synthesized, and the anti-tumor activities were evaluated.

GLUT1, a member of the mammalian facilitative glucose transporter family [47, 48], is significantly overexpressed in many human cancers, for example, non-small cell lung cancer [49], breast cancer [50], gastric carcinoma [51], pancreatic carcinoma [52] and colorectal cancer [53]. In this research we compared GLUT1 protein expression in six cancer cell lines, analyzed their different sensitivity in anti-proliferation activity of sugar conjugates and raised a hypothesis that the transportation of sugar conjugated platinum complexes might be mediated by GLUT1. As summarized in Table 2, the cytotoxicities of different sugar conjugated platinum(II) complexes 5a, 5b and 5c are exhibited positive correlation with the expression level of GLUT1 in the most overexpressing HT29 cell lines. For the lowest GLUT1 expression MCF-7 cell line, the glucose and mannose conjugated platinum(II) complexes 5a and 5b still showed high sensitivities but the galactose conjugated compound 5c exhibited weaker cytotoxicity. This phenomenone needs to be further investigated as other subtypes of GLUTs may overexpressing in MCF-7 cells and 5c may have different cellular uptake compared to 5a and 5b.

To test the hypothesis, three different assays on cellular uptake mechanism were performed. Results from the inhibitor dependent cytotoxicity assay of all three sugar conjugates and oxaliplatin revealed that the cell-killing potency of sugar conjugates had significant dependency on quercetin, whereas oxaliplatin did not exhibit quercetin dependent cytotoxicity. In consistence with the inhibitory effect of quercetin, improvement of the IC50 values of sugar conjugates for anti-tumor activity against HT29 were also observed when GLUT1 expression was down-regulated by short-hairpin RNAs (shRNA). However, the anti-tumor efficacy of oxaliplatin showed no significant difference between HT29-mock and HT29-shRNA cells. To further confirm our hypothesis, the intracellular platinum accumulation assay was performed. As expected, the platinum uptake of compound 5a in HT29 and HT29-mock cells was greater than that in HT29-shRNA cells. Based on the results, both RNAi knockdown and the inhibitor blockade of the GLUT1 resulted in decreased cytotoxicity and decreased intracellular platinum accumulation in HT29 cell line, suggesting that the sugar conjugates was transported mainly via GLUT1 and all three sugars as glycoconjugation motif have similar effect to enable the platinum(II) complexes as substrate for GLUT1 mediated tumor targeting.

With the aim to understand the intrinsic reactivity of the interactions of platinum complexes with N-bonding ligands, we investigated the complex-formation kinetics of 5a, 5b, 5c with nitrogen donor ligands 5′-GMP implementing ^1^H NMR analysis to mimic the Pt(II)-DNA adduct formation and compared to that of oxaliplatin. It is well known that 5′-GMP can coordinate to metal ions through the N1 and N7 positions, but only bind through the N7 position in a neutral medium [35]. Therefore, nucleophilic substitution reaction with 5′-GMP occurs at the N7 atom of purine base. Downfield shift of H8 proton on 5′-GMP from hydrogen spectrum can be used to monitor the newly formed platinum(II)-GMP complex. Our 5′-GMP consumption kinetics results indicated that 5a, 5b and 5c exhibited faster reactions with nucleophlic base 5′-GMP compared to oxaliplatin. The intrinsic reactivity investigation from 5′-GMP reaction kinetics study demonstrated that the 2-flouromalonato-platinum(II) glycoconjugates derived from glucose, mannose and galactose as alternative sugar motif have greater apoptotic potential compared to oxaliplatin. Researches on mechanism of cisplatin and oxaliplatin have revealed that platinum-DNA adduct formation based on intrastrand or interstrand chelation involving two adjacent guanines, interferes with cell division, replication and triggers apoptosis [54, 55]. It has been reported that cisplatin and oxaliplatin form approximately 60–65% intrastrand adducts at the GG sites on the DNA [6, 56, 57]. The intrastrand crosslinks to d(GpG) is the most abundant species in cisplatin-damaged DNA. Agarose gel electrophoresis was used to investigate the ability of sugar conjugates to bind and unwind DNA from the supercoiled form of pUC18 plasmid DNA to the open circular or linear forms. Results from DNA unwinding assay indicated that when pUC18 plasmid DNA was treated with sugar conjugates, cisplatin and oxaliplatin, the mobility of supercoiled forms and open circular form plasmid DNA bands increased and showed concentration dependency. The change in mobility of pUC18 plasmid DNA bands caused by sugar conjugated platinum complexes is believed to be due to interstrand binding to the GG sites of DNA. pUC18 plasmid DNA could be digested with restriction enzyme BamHI at the specific GG site to construct linear form III DNA [37]. All concentrations of the compounds prevented BamHI digestion in varying degrees. The prevention of BamH1 digestion showed correlation with the increasing concentration of platinum complexes. The interaction observed for the platinum(II)-sugar conjugates with plasmid (pUC18) DNA, along with the BamHI digestion of the mixtures point to the formation of Pt-GG intrastrand DNA binding adducts, which is similar to that of oxaliplatin.

Compared with oxaliplatin, compound 5a showed an improved potency and safety profiles in ascetic leukemia L1210 bearing DBA/2 mice model. Unlike our previous work [24], in which compound 5a and oxaliplatin were administered at the equitoxic doses of 70% of MTD. In this work, a reduced dose of 18% MTD of 5a (11 mg/kg), which is equal molar with oxaliplatin (7 mg/kg), was administered in ascetic leukemia L1210 bearing DBA/2 mice model. With regard to its anti-leukemic activity, compound 5a was still able to extend the mean life span, indicating improved safety on anti-leukemic efficacy. In addition, compound 5a suppresses tumor growth in HT29 colon cancer xenografts mice model. Compared to the vehicle-treated control animals, experimental animals treated with compound 5a resulted in a clear suppression of tumor growth. As compared with oxaliplatin, significant suppress on tumor growth inhibition (TGI) and tumor weight of 5a was observed. In addition, 100% maximal tolerated dose of oxaliplatin was applied in this experiment, whereas 5a was applied at 70% maximal tolerated dose. These results indicated that 5a was even more effective in inhibiting tumor growth in the HT29 xenograft mouse models than oxaliplatin, which were associated with a significant reduction in colon cancer cell proliferation.

In summary, based on Warburg effect tumor targeting strategy, a series of fluorine substituted platinum(II)-sugar conjugates were designed and synthesized to leverage the GLUT-mediated tumor specific drug uptake. The introduction of fluorine and sugar moiety in the malonato leaving ligand tremendously improved water solubility of the platinum(II) complexes. In addition to the *in vitro* and *in vivo* anti-tumor activity evaluations, we systematically revealed the cellular transport mechanism of the sugar conjugated fluorine containing platinum complexes by utilizing different assessment strategies. Recent studies have demonstrated the drug-induced neurotoxicity of oxaliplatin are due to its drug uptake mechanism with certain type of transporters such as organic cation transporters (OCTs), which are ubiquitously expressed in normal cells and neuron system [58–60]. Compare with oxaliplatin, the glucose, mannose and galactose conjugated highly water soluble (trans-*R,R*-cyclohexane-1,2-diamine)-2-flouromalonato-platinum(II) complexes exhibited not only comparable or better *in vitro* cytotoxicities, but also improved potency and safety in both the L1210 bearing ascitic leukemia and human colon cancer xenograft animal models. These results provide evidences and prove the advantages for glucose transporter mediated tumor specific drug delivery and the Warburg effect mediated tumor targeting. Both *in vitro* and *in vivo* results from the current proof-of-concept study support that the glucose conjugated compound 5a has great potential for further pre-clinical evaluations as Warburg effect targeted anticancer agent.

## MATERIALS AND METHODS

### Materials and instruments

All reagents and solvents were of analytical grade or HPLC grade purchased from commercial suppliers and used without further purification. High purity water was obtained from a Milli-Q water system (18.2 MΩ cm, Milli-Q Advantage, Darmstadt, Germany). ^1^H and ^13^C NMR spectra were recorded with a Bruker Avance 600 MHz NMR spectrometer at 600 (^1^H) and 150.93 (^13^C). All chemical shift values are reported as δ ppm using CDCl_3_ or D_2_O as solvent, unless otherwise stated. Infrared spectra were recorded with a Bruker Tensor 27 FT-IR spectrometer. High resolution mass spectra were measured on a Bruker MicroTOF spectrometer. HPLC analyses were performed with CXTH-LC3000 analytical and semi-preparative gradient HPLC system using Cosmosil PAQ-C18 (4.6 × 250 mm, 5 μm) and DaisoGel C18 (20 × 250 mm, 10 μm) column respectively at ambient temperature. The following solvent system was used: H_2_O and MeOH/0.1% formic acid, at a flow rate of 1 mL/min for analytical and 10 mL/min for preparative purification. For target compounds studied in cancer cells and DNA binding experiments, an analytical purity of > 95% was confirmed using this setup. MTS assay were performed on Thermo Scientific Multiskan^®^ Spectrum (Thermo Fisher Scientific Inc., USA). Electrophoresis experiments were carried out in a Bio-Rad Mini subcell GT horizontal electrophoresis system. Photographs of the gels were taken with a Chemidoc XRS & (Fire Wire) Camera (Chemidoc XRS Chemiluminescent Gel Documentation Cabinet). Quantitative real-time PCR assay was performed on LightCycler® 96 Instrument (Roche Life Science, UK). Platinum concentration determination was performed using inductively-coupled plasma mass spectrometry by Agilent 7700X ICP-MS (Agilent Technologies, Tokyo, Japan).

### Chemistry

1-O-(2, 3, 4, 6-tetraacetyl-α-D-glucoside)-2-bromoethane (1a) 1a as a known compound was prepared by following the literature method [30].

1-O-(2, 3, 4, 6-tetraacetyl-α-D-mannoside)-2-bromoethane (1b) As a known compound, penta-O-acetyl-α-D-mannopyranose was prepared according to the literature procedure [29, 30]. A solution of 4.69 g (12.0 mmol) of penta-O-acetyl-α-D-mannopyranose and 2.46 g (19.7 mmol) of ethylene bromohydrin in 48 mL of dichloromethane was put in a 250 mL round bottom flask in an argon atmosphere. Subsequently, 1.8 mL of boron trifluoride diethyl ether complex was added dropwise. The reaction mixture was continuously stirred in ice bath for one hour and then warmed up to room temperature and stirred overnight. 200 mL of DCM was added to the reaction solution and the organic phase was washed with water (150 mL), saturated sodium hydrogen carbonate aqueous solution (200 mL) and saturated saline (200 mL). The organic solution was dried over anhydrous sodium sulfate, filtered and evaporated under vaccum to give the crude product. The pure 1b was obtained as solid by recrystallization from ethanol in 55% yield (3.0 g). ^1^H NMR (600 MHz, CDCl_3_) δ 5.36 – 5.27 (m, 3H), 4.87 (d, *J* = 1.5 Hz, 1H), 4.27 (dd, *J* = 12.7, 5.9 Hz, 1H), 4.15 – 4.12 (m, 2H), 3.99 – 3.87 (m, 2H), 3.51 (t, *J* = 6.0 Hz, 2H), 2.16 (s, 3H), 2.10 (s, 3H), 2.05 (s, 3H), 1.99 (s, 3H); ^13^C NMR (151 MHz, CDCl_3_) δ 170.29, 169.71, 169.59, 169.53, 97.57, 69.21, 68.88, 68.83, 68.30, 65.82, 62.26, 29.79, 20.66, 20.55, 20.53, 20.48; LRMS (ESI) *m/z* 455.0 (M +H^+^), 457.0 (M+2+H^+^).

1-O-(2, 3, 4, 6-tetraacetyl-β-D-galactoside)-2-bromoethane (1c) 1c in the form of solid was prepared according to the procedure of 1b from penta-O-acetyl-β-D-galactopyranose. Yield: 75%; ^1^H NMR (600 MHz, CDCl_3_) δ 5.38 (d, *J* = 2.9 Hz, 1H), 5.22 (dd, *J* = 10.4, 8.0 Hz, 1H), 5.01 (dd, *J* = 10.5, 3.4 Hz, 1H), 4.52 (d, *J* = 8.0 Hz, 1H), 4.18 – 4.09 (m, 3H), 3.91 (t, *J* = 6.6 Hz, 1H), 3.83 – 3.79 (m, 1H), 3.47 – 3.44 (m, 2H), 2.14 (s, 3H), 2.07 (s, 3H), 2.04 (s, 3H), 1.97 (s, 3H); ^13^C NMR (151 MHz, CDCl_3_) δ 170.29, 169.71, 169.59, 169.53, 97.57, 69.21, 68.88, 68.83, 68.30, 65.82, 62.26, 29.79, 20.66, 20.55, 20.53, 20.48; LRMS (ESI) *m/z* 455.0 (M +H^+^), 457.0 (M+2+H^+^).

2,3,4,6-Tetra-O-acetyl-α-D-glucopyranosyl-2- malonatediethylester (2a) To a solution of 1a (2.21 g, 4.9 mmol) in dry DMF (10 mL) was added diethylmalonate (1.03 g, 6.5 mmol) followed by K_2_CO_3_ (1.83 g, 13.2 mmol). The reaction mixture was stirred at room temperature overnight. Completion of the reaction was monitored by TLC (petroleum/ethyl acetate = 3:1). 60 mL of ethyl acetate was added to the reaction mixture and stirred for 10 min. then the mixture was poured into 100 mL of saturated NH_4_Cl aqueous solution. The organic layer was washed with brine (50 mL) and water (30 mL x 2), dried over MgSO_4_. The solvent was removed under vaccum, and the residue was purified by column chromatography (PE:EA=3:1) to give 2a (2.35 g, 88.0%) as a yellow oil. ^1^H NMR (400 MHz, CDCl_3_): δ 5.41 (t, *J* = 10.0 Hz, 1H), 4.97-5.06 (m, 2H), 4.83 (dd, *J* = 4.0 Hz, 8.0 Hz, 1H), 4.09-4.26 (m, 5H), 4.05 (d, *J* = 12.0 Hz, 1H), 3.98 (d, *J* = 12.0 Hz, 1H), 3.73-3.80 (m, 1H), 3.47-3.56 (m, 1H), 3.36-3.43 (m, 1H), 2.11-2.22 (m, 2H), 2.09 (s, 3H), 2.04 (s, 3H), 2.00 (s, 3H), 1.98 (s, 3H), 1.22-1.28 (m, 6H); ^13^C NMR (100 MHz, CDCl_3_): *δ* 170.50, 170.08, 169.97, 169.48, 168.91, 168.85, 100.88, 96.86, 70.64, 70.04, 67.24, 65.69, 61.73, 61.45, 60.25, 48.72, 28.41, 20.90, 20.58, 20.50, 13.98, 13.96; HRMS: Calcd. for C_23_H_34_O_14_ (M^+^ + 1): 535.1949, found: 535.1947.

2,3,4,6-tetra-O-acetyl-α-D-mannopyranosyl-2-malonatediethylester (2b) To an ice-cooled solution of 1b (3.35 g, 7.4 mmol) and diethylmalonate (1.59 g, 9.9 mmol) in dry DMF (18 mL) was added potassium carbonate (2.73 g, 19.8 mmol) followed by potassium iodide(2.1 g, 12.6 mmol). Then the reaction mixture was stirred at 50°C overnight. After completion of the reaction (monitored by TLC, petroleum/ethyl acetate = 3:1), ethyl acetate (100 mL) was added. The organic layer washed with saturated NH_4_Cl (aq.) (50 mL), followed by brine (100 mL) and water (100 mL), dried over anhydrous sodium sulfate and concentrated under reduced pressure. The residue was purified by silica gel column chromatography (PE:EA=4:1) to give 2b (3.65 g, 92.8%) as a white solid. ^1^H NMR (600 MHz, CDCl_3_) δ 5.27 – 5.21 (m, 3H), 4.76 (d, *J* = 1.0 Hz, 1H), 4.28 – 4.18 (m, 5H), 4.08 (dd, *J* = 12.2, 2.2 Hz, 1H), 3.97 – 3.95 (m, 1H), 3.78 – 3.74 (m, 1H), 3.52 – 3.49 (m, 2H), 2.28 – 2.16 (m, 2H), 2.13 (s, 3H), 2.09 (s, 3H), 2.03 (s, 3H), 1.97 (s, 3H), 1.26 (t, *J* = 6.8 Hz, 6H); ^13^C NMR (151 MHz, CDCl_3_) δ 169.98, 169.38, 169.26, 169.22, 168.59, 168.56, 97.32, 68.97, 68.78, 68.31, 65.66, 65.29, 61.09, 46.75, 27.97, 20.32, 20.20, 20.14, 13.66; LRMS (ESI) *m/z* 535.2 (M +H^+^).

2,3,4,6-tetra-O-acetyl-β-D-galactopyranosyl-2-malonatediethylester (2c) 2c was prepared according to the procedure of 2b from 1-O-(2, 3, 4, 6-tetraacetyl-β-D-galactoside)-2-bromoethane as a white solid. Yield: 93%; ^1^H NMR (600 MHz, CDCl_3_) *δ* 5.20 (s, 1H), 4.98 (t, *J* = 9.0 Hz, 1H), 4.84 (d, *J* = 10.2 Hz, 1H), 4.30 (d, *J* = 7.8 Hz, 1H), 4.02 – 3.95 (m, 6H), 3.78 (d, *J* = 5.1 Hz, 2H), 3.37 – 3.32 (m, 2H), 2.08 – 1.97 (m, 5H), 1.91 (s, 3H), 1.86 (s, 3H), 1.79 (s, 3H), 1.09 – 1.07 (m, 6H); ^13^C NMR (151 MHz, CDCl_3_) δ 170.17, 170.07, 169.89, 169.34, 168.92, 168.90, 100.94, 70.72, 70.42, 68.54, 66.92, 66.79, 61.26, 61.24, 61.13, 48.12, 28.48, 20.54, 20.46, 20.38, 13.93, 13.87; LRMS (ESI) *m/z* 535.2 (M +H^+^).

2,3,4,6-tetra-O-acetyl-α-D-glucopyranosyl-2-fluoromalonatediethylester (3a) To an ice-cooled solution of 2a (2.10 g, 3.9 mmol) in dry DMF (10 mL) was added NaH (0.19 g, 4.8 mmol, 60% suspension in mineral oil) and stirred for 30 min. Then a solution of Selectfluor (1.72 g, 4.8 mmol) in 3 mL of dry DMF was added to the ice-cooled reaction mixture and stirred overnight at room temperature. The reaction solution was poured into ice water (15 mL) and extracted with 50 mL of ethyl acetate. The organic layer was washed successively with satd. NH_4_Cl (15 mL), brine (15 mL) and water (15 mL), dried over MgSO_4_ and evaporated under vaccum to dryness. The resulting oil was subjected to column chromatography (PE:EA = 3:1) to give 3a as a white solid (2.0 g, 91.0%). ^1^H NMR (400 MHz, CDCl_3_): δ 5.35 (t, *J* = 10.0 Hz, 1H), 4.96-5.10 (m, 2H), 4.75-4.85 (m, 1H), 3.75-4.45(m, 9H), 3.50-3.60 (m, 1H), 2.57 (t, *J* = 6.0 Hz, 1H), 2.52 (t, *J* = 6.0 Hz, 1H), 2.08 (s, 6H), 2.02 (s, 3H), 1.99 (s, 3H), 1.30 (t, *J* = 6.0 Hz, 6H); ^13^C NMR (100 MHz, CDCl_3_): δ 170.58, 170.19, 169.91, 169.57, 166.06, 165.89, 165.78, 165.61, 95.93, 95.68, 92.79, 91.48, 70.70, 69.96, 68.45, 68.32, 67.32, 65.76, 62.74, 61.80, 61.53, 48.77, 33.71, 33.57, 28.47, 20.64, 20.61, 20.57, 14.01, 14.02, 13.94, 13.92; HRMS: Calcd. for C_23_H_33_FO_14_ (M^+^ + 1): 553.1854, found: 553.1857.

2,3,4,6-tetra-O-acetyl-α-D-mannopyranosyl-2-fluoromalonatediethylester (3b) Sodium hydride (0.211 g, 5.3 mmol, 60% suspension in mineral oil) was added to an ice-cooled solution of 2b (2.35 g, 4.4 mmol) in dry DMF (14 mL) under a nitrogen atmosphere. The reaction mixture was stirred at 0°C for 30 min. Then selectfluor (1.87 g, 5.3 mmol) was added. The mixture was slowly warmed up to room temperature and stirred overnight. When the reaction was complete, 45 mL of ice water and 45 mL of ethyl acetate was added to extract the product. The organic phase was washed with sat. NH_4_Cl (15 mL), water (15 mL) and brine (15 mL), dried over MgSO_4_ and evaporated under reduced pressure. The resulting oil was subjected to silica gel chromatography (PE:EA = 4:1) to afford 3b as a colorless oil (2.01 g, 82.7%). ^1^H NMR (600 MHz, CDCl_3_) δ 5.27 – 5.17 (m, 3H), 4.74 (s, 1H), 4.37 – 4.25 (m, 5H), 4.10 (d, *J* = 12.1 Hz, 1H), 4.04 – 4.01 (m, 1H), 3.94 – 3.90 (m, 1H), 3.58 – 3.55 (m, 1H), 2.63 – 2.57 (m, 2H), 2.13 (s, 3H), 2.09 (s, 3H), 2.04 (s, 3H), 1.96 (s, 3H), 1.32 – 1.29 (m, 6H); ^13^C NMR (151 MHz, CDCl_3_) δ 170.20, 169.55, 169.49, 165.89, 165.72, 165.50, 97.55, 92.58, 91.27, 68.93, 68.82, 68.49, 65.57, 62.51, 62.13, 61.46, 33.29, 33.15, 20.47, 20.39, 20.36, 20.29, 13.72, 13.69; LRMS (ESI) *m/z* 553.1 (M +H^+^).

2,3,4,6-tetra-O-acetyl-β-D-galactopyranosyl-2-fluoromalonatediethylester (3c) 3c was prepared according to the procedure of 3b from 2,3,4,6-tetra-O-acetyl-β-D-galactopyranosyl-2-malonatediethylester as a colorless oil. Yield: 82%; ^1^H NMR (600 MHz, CDCl_3_) δ 5.37 (s, 1H), 5.14 (t, *J* = 9.2 Hz, 1H), 4.98 (dd, *J* = 10.4, 3.0 Hz, 1H), 4.46 (d, *J* = 8.0 Hz, 1H), 4.30 – 4.12 (m, 7H), 3.89 (t, *J* = 6.5 Hz, 1H), 3.68 (dd, *J* = 16.1, 7.6 Hz, 1H), 2.60 – 2.57 (m, 2H), 2.15 (s, 3H), 2.10 (s, 3H), 2.06 (s, 3H), 1.97 (s, 3H), 1.31 – 1.28 (m, 6H); ^13^C NMR (151 MHz, CDCl_3_) δ 170.22, 170.08, 169.92, 165.76, 165.67, 165.60, 100.93, 92.95, 91.64, 70.99, 70.60, 68.39, 66.98, 63.61, 62.60, 62.44, 61.16, 33.88, 33.74, 20.70, 20.52, 20.50, 20.41, 13.81; LRMS (ESI) *m/z* 553.1 (M +H^+^).

2-fluoromalonic acid-α-D-glucopyranosyl conjugate (4a) To a solution of 3a (2.20 g, 4.0 mmol) in MeOH (10 mL) at room temperature. A solution of NaOH (1.44 g, 36.0 mmol) dissolved in 30 mL of water was then added to the reaction mixture. After stirred at 90°C for 8h, the reaction solution was passed through a short column of DOWEX 88 strong acid cation resin and the desired product 4a was obtained as a white solid after prep-HPLC purification and lyophilization (5% MeOH-95% H_2_O-0.1% HCOOH): (0.91 g, 69.4%). ^1^H NMR (400 MHz, D_2_O): δ 4.63 (d, J = 4.0 Hz, 1H), 3.65-3.75 (m, 1H), 3.55-3.65 (m, 1H), 3.45-3.55 (m, 1H), 3.35-3.45 (m, 3H), 3.20-3.30 (m, 1H), 3.15 (t, J = 10.0 Hz, 1H), 2.26-2.56 (m, 2H); ^13^C NMR (100 MHz, D_2_O): δ 169.99, 169.88, 169.73, 169.62, 98.35, 94.25, 92.29, 72.96, 71.78, 71.32, 69.46, 62.07, 60.47, 34.00, 33.79; HRMS: Calcd. for C_11_H_17_FO_10_ (M^+^-1): 327.0806, found: 327.0809.

2-fluoromalonic acid-α-D-mannopyranosyl conjugate (4b) To the solution of 3b (1.1 g, 2.0 mmol) in MeOH (5 mL) was added a solution of NaOH (0.72 g, 18.0 mmol) in water (20 mL) at room temperature. Then the reaction mixture was stirred at 90°C for 8 hours. The reaction was monitored with HPLC. Upon completion of the reaction, the reaction solution was passed through a short column of DOWEX 88 strong acid cation resin. After prep-HPLC purification and lyophilization, the desired product 4b was obtained as a white solid (3% MeOH-97% H_2_O-0.1% HCOOH): (0.49 g, 74.3%). ^1^H NMR (600 MHz, D_2_O) *δ* 4.70 (s, 1H, α – H1), 3.84 – 3.77 (m, 3H), 3.67 – 3.53 (m, 5H), 2.50 – 2.43 (m, 2H); ^13^C NMR (151 MHz, D_2_O) *δ* 170.34, 170.22, 170.08, 169.96, 99.74 (α – C1), 94.13, 92.19, 72.66, 70.41, 69.70, 66.53, 61.45, 60.81, 33.83; HRMS: Calcd. for C_11_H_17_FO_10_ (M^+^-1): 327.0806, found: 327.0810.

2-fluoromalonic acid-β-D-galactopyranosyl conjugate (4c) 4c was prepared according to the procedure of 4b from *2,3,4,6-tetra-O-acetyl-β-D-galactopyranosyl-2-fluoromalonatediethylester* as a white solid. Yield: 67%; ^1^H NMR (600 MHz, D_2_O) δ 4.33 (d, *J* = 7.8 Hz, 1H, β – H1), 4.06 – 4.04 (m, 1H), 3.88 (s, 1H), 3.77 – 3.60 (m, 5H), 3.45 (t, *J* = 8.8 Hz, 1H), 2.52 (d, *J* = 22.8 Hz, 2H); ^13^C NMR (151 MHz, D_2_O) δ 169.59, 169.42, 169.39, 169.22, 102.50 (β – C1), 93.65, 92.35, 74.63, 72.32, 70.52, 68.30, 63.70, 60.65, 33.87, 33.73; HRMS: Calcd. for C_11_H_17_FO_10_ (M^+^-1): 327.0806, found: 327.0809.

(trans-R,R-cyclohexane-1,2-diamine)-2-fluoromalonatoplatinum(II)-α-D-glucose conjugate (5a) To a solution of 4a (0.30 g, 1.0 mmol) in 20 mL of water under an argon atmosphere was slowly added a solution of Ba(OH)_2_·8H_2_O in 10 mL of water until pH = 8.0. The mixture was stirred for 30 min at room temperature. [trans-*R*, *R*-cyclohexane-1,2-diamine]PtSO_4_ (0.49 g, 1.2 mmol) in 10 mL of water was then added to the reaction mixture and stirred in the dark overnight. The precipitate was filtered and the filtrate was subjected to prep-HPLC purification and lyophilization (20% MeOH-80% H_2_O) to afford 5a as a white solid. Yield: 0.40 g (58.3%); ^1^H NMR (400 MHz, D_2_O): δ 4.86 (d, *J* =4.0 Hz, 1H), 3.85-4.00 (m, 2H), 3.60-3.85 (m, 4H), 3.40-3.60 (m, 2H), 3.25-3.35 (m, 2H), 2.31 (br, 2H), 1.96 (d, *J* = 8.0 Hz, 2H), 1.48 (d, *J* =8.0 Hz, 2H), 1.10-1.30 (m, 2H), 0.90-1.10 (m, 2H); ^13^C NMR (100 MHz, D_2_O): δ 175.57, 175.41, 175.22, 175.06, 98.58, 95.88, 94.58, 72.96, 72.13, 71.46, 69.64, 62.83, 62.59, 62.36, 60.63, 37.36, 37.20, 31.80, 31.63, 23.88, 23.80; HRMS: Calcd. for C_17_H_29_FN_2_O_10_Pt (M^+^+Na): 658.1346, found: 658.1348.

(trans-R,R-cyclohexane-1,2-diamine)-2-fluoromalonatoplatinum(II)-α-D-mannose conjugate (5b) To a solution of 4b (0.34 g, 1.1 mmol) in water (20 mL) under argon atmosphere was slowly added an aqueous solution of Ba(OH)_2_·8H_2_O until the pH = 8.0. The mixture was stirred at room temperature for 30 min. [trans-R, R-cyclohexane-1,2-diamine]PtSO4 (0.54 g, 1.3 mmol) in 10 mL of water was then added and the reaction mixture was stirred in the dark overnight. After completion of the reaction, the solution was filtered and the filtrate was subjected to preparative HPLC for purification. The platinum(II) complex 5b was obtained after lyophilization as a white solid. Yield: 0.49 g (70.2%); ^1^H NMR (600 MHz, D_2_O) δ 4.92 (s, 1H, α – H1), 3.98 – 3.59 (m, 9H), 2.44 – 2.32 (m, 3H), 2.02 (d, *J* = 11.3 Hz, 2H), 1.56 (d, *J* = 9.3 Hz, 3H), 1.32 – 1.10 (m, 4H); ^13^C NMR (151 MHz, D_2_O) δ 175.77, 175.68, 175.61, 175.51, 99.88 (α – C1), 95.98, 94.67, 72.81, 70.48, 69.78, 69.72, 66.67, 62.03, 61.92, 61.75, 60.89, 37.63, 37.49, 30.76, 23.38; HRMS: Calcd. for C_17_H_29_FN_2_O_10_Pt (M^+^+Na): 658.1346, found: 658.1339.

(trans-R,R-cyclohexane-1,2-diamine)-2-fluoromalonatoplatinum(II)-β-D-galactose conjugate (5c) 5c was prepared according to the procedure of 5b from *2-fluoromalonic acid-β*-*D-galactopyranosyl conjugate* as a white solid. Yield: 69%; ^1^H NMR (600 MHz, D_2_O) *δ* 4.43 (d, *J* = 7.7 Hz, 1H, β – H1), 4.18 (d, *J* = 5.2 Hz, 1H), 4.00 – 3.65 (m, 7H), 3.54 – 3.49 (m, 2H), 2.44 (s, 2H), 2.03 (dd, *J* = 20.4, 12.3 Hz, 2H), 1.56 (d, *J* = 10.8 Hz, 2H), 1.35 – 1.19 (m, 2H), 1.19 – 0.97 (m, 2H); ^13^C NMR (151 MHz, D_2_O) *δ* 175.45, 175.29, 174.95, 174.79, 102.81 (β – C1), 95.86, 94.56, 75.16, 72.58, 70.97, 68.60, 64.42, 62.65, 61.05, 36.79, 36.64, 31.86, 31.57, 23.86; HRMS: Calcd. for C_17_H_29_FN_2_O_10_Pt (M^+^+Na): 658.1346, found: 658.1335.

### Water solubility analysis

Water solubility analysis was performed following the method we described before [32].

### Cell lines and cell culture conditions

The human colon cancer (HT29), breast cancer (MCF7), prostate cancer (DU145) and ovarian cancer (SK-OV-3) cell lines, were obtained from Central Institute of Pharmaceutical Research, CSPC Pharmaceutical Group, China. The human lung cancer cell lines H460 and A549 were purchased from the American Type Culture Collection (ATCC). HT29cisR, the cisplatin-resistant counterpart to HT29, was performed according to a procedure published in the literature [33, 61]. HT29cisR obtained by this selection procedure were used in this study and cisplatin (1 μM) was added every third passage to maintain its resistance. MCF7 and SK-OV-3 were cultured in advanced Dulbecco's modified Eagle medium (DMEM), and other cell lines were cultured as adherent monolayer in RPMI-1640 supplemented with 1% (w/v) glutamine and 10% (v/v) fetal bovine serum (Gibco) at 37°C under a humidified atmosphere containing 5% CO_2_ and 95% air.

### Cytotoxicity assays

Cytotoxicity assays were carried out by the Celltiter 96 aqueous nonradioactive cell proliferation assay (Promega, Madison, WI). Cells were harvested by trypsinization and seeded in a 96-well flat-bottomed microplate in volumes of 100 μL at the following appropriate densities: 5.0 × 10^3^ (A549), 8.0 × 10^3^ (HT29), and 3.0 × 10^3^ (H460), 5.0 × 10^3^ (SKOV3), 8.0 × 10^3^ (MCF7), 8.0 × 10^3^ (DU145) cells per well. After being allowed to settle and resume exponential growth for 24 h, the cells were treated either with vehicle or with appropriate serial dilutions of the complexes. Stock solutions of sugar conjugated platinum complexes 5a-5c and oxaliplatin were dissolved in deionized water. The cisplatin stock solution was prepared in PBS. All the tested compounds were dissolved at room temperature prior to use and serially diluted in medium. Cells were incubated in a humidified atmosphere of 5% CO_2_ at 37°C for 72h. After incubation, 20 μL of MTS/PMS solution were added to each well and the mixtures were allowed to equilibrate for 2 h. The absorbance of MTS products was measured spectrophotometrically at 490 nm with microplate reader (VERSAmax, USA). The IC_50_ values of each test sample were calculated from nonlinear curve fits using dose-response equation in GraphPad Prism (La Jolla, CA). Experimental conditions were performed in five replicates and the calculated IC_50_ values are averages of three individual experiments.

### Stable transfection

Two sets of oligonucleotides encoding SLC2A1-specific shRNAs were designed to clone it into the pLKO.1 vector (BD Biosciences Clontech). The plasmids shGLUT1-1 and shGLUT1-2 was verified by DNA sequencing. Lentiviruses containing SLC2A1-specific shRNA in pLKO.1 plasmid were generated using 293T cells as packaging cell lines. HT29 cells were seeded in 6-well plates at a density of 5×10^5^ cells per well. After 24-h incubation, virus supernatant was incubated on target cells for 6 hours with 1 μg/mL polybrene. Forty-eight hours after transfection, cells were treated with puromycin (Solarbio) to select clones with stable expression of shGLUT1-1 and shGLUT1-2.

Target sequence was shGLUT1-1

forword: 5′-CCGGCTTCAAAGTTCCTGAGACTAACTCGAGTTAGTCTCAGGAACTTTGAAGTTTTTG-3′

Reverse:

5′-AATTCAAAAACTTCAAAGTTCCTGAGACTAACTCGAGTTAGTCTCAGGAACTTTGAAG-3′

shGLUT1-2

forword: 5′-CCGGCTCCAACTGGACCTCAAATTTCTCGAGAAATTTGAGGTCCAGTTGGAGTT-3′

Reverse: 5′-AATTCAAAAACTCCAACTGGACCTCAAATTTCTCGAGAAATTTGAGGTCCAGTT-3′

pLKO shRNAs targeting GLUT1 were from the RNAi consortium at the Broad Institute.

### Real-time PCR

Cells in culture dish were directly lysed by adding TRIZOL® Reagent. RNA was extracted with total RNA isolation kit (Solarbio) according to manufacturer's protocol.

Total RNA suspended in RNase-free water was quantified by Nanodrop ND8000 (Thermo Scientific) and used as the template for reverse transcription. cDNA was prepared from 1 μg RNA with the RevertAid RT Reverse Transcription Kit (Invitrogen™) using oligo-dT primers, following manufacturer's instructions. The cDNA was diluted 10 times and 2 μL were used for each reaction. qRT-PCR was performed on 7500 fast RealTime PCR (Roche) by using SYBR Green Real-Time PCR Master Mixes (Thermo Scientific™), according to the manufacturer's protocol. Reactions were performed under the following conditions: enzyme activation for 10 minutes at 95°C, followed by 40 cycles of denaturation for 15 s at 95°C and 60 s at 60°C. A melting cycle consisting of 95°C for 10 s, 65°C for 60 s, 97°C for 1 s and data collection for 30 seconds at 37°C.

Experiments were repeated three times, and the GLUT1 mRNA level was normalized to GAPDH. Primers: GLUT1 (Forward Primer- GGCCAAGAGTGTGCTAAAGAA Reverse Primer- ACAGCGTTGATGCCAGACAG) GAPDH (Forward Primer-GGAGCGAGATCCCTCCAAAAT Reverse Primer- GGCTGTTGTCATACTTCTCATGG)

### Western blot

Cells were washed twice with ice-cold 1 × PBS and lysed using RIPA lysis buffer (Sigma) supplemented with cOmplete, Mini Protease Inhibitor Tablets (1 per 10mL) (Roche) and PhosSTOP phosphatase inhibitor tablets (1 per 10mL). Lysates were sonicated and then centrifuged at 12000 rpm at 4°C for 10 min. Total protein concentration was quantified with pierce BCA protein assay kit (Thermo Scientific™).10 μg of total cell protein were loaded onto 10% SDS-PAGE and then transferred to a microporous polyvinylidene difluoride (PVDF) membrane. Western blotting was performed using Polyclonal anti-GLUT1 antibody (1:2000) (ab652, Abcam), anti-β-actin antibody (Sigma) and goat anti-rabbit or goat anti-mouse IgG-peroxidase conjugate as the secondary antibody. The detection of the proteins was made using chemiluminescence substrate.

### Determination of the platinum concentration in cells

HT29 cells were seeded in 10 cm dishes at densities of 6 × 10^6^ cells/dish and kept at 37°C in a humidified 5% CO_2_ incubator. After 2 h incubation with complete medium containing 200 μM of platinum complex 5a, the medium was removed, and the cells were washed with ice-cold PBS, trypsinized, and resuspended in 1 mL of PBS. The cell suspensions were centrifuged at 2500 rpm for 10 min and remove the PBS. Vortex vigorously to resuspend cells. Nuclei lysis solution was added to lyse the cells. DNA was extracted with TIANamp Genomic DNA kit (TIANGEN). The extracted DNA was dissolved in concentrated nitric acid (0.05 mL) and diluted with 4.95 mL of deionized water (final concentration 1% v/v) for determining the platinum content. The platinum content taken up by the cells was determined by Agilent 7700X ICP-MS (Agilent Technologies, Tokyo, Japan). Calibrations were done using standard solutions containing 6.25, 12.5, 25, 50 and 100 ppb platinum.

### Reaction of sugar conjugated platinum complexes with 5′-Guanosine monophosphate

Kinetic study of the substitution reactions between sugar conjugated platinum complexes with 5′-Guanosine Monophosphate was performed on Bruker Avance 400 MHz spectrometer by ^1^H NMR analysis. Platinum complex (5 mM) and 5′-GMP (10 mM) were freshly prepared in deuterium oxide (D_2_O) at 37°C. A 5 mM solution of the NaCl in D_2_O was added to the mixture just before running the spectra. All samples were incubated at 37°C in a water bath to simulate physiological conditions and 1H NMR spectra were recorded at 295 K over a period of several days.

### Interaction with plasmid (pUC18) DNA

Solutions of pUC18 plasmid DNA (50 μg/mL) were incubated with increasing concentrations of cisplatin, oxaliplatin and compounds 5a-5c ranging from 12.5 to 100 μM in water bath at 37°C for 4 h in the dark. pUC18 plasmid DNA treated with buffer (50 mM Tris-HCl and 100mM NaCl) was prepared as negative control. After the incubation period, drug-DNA mixtures were loaded onto 0.8% agarose gel and electrophoresis was performed in Tris-acetate/EDTA buffer (TAE) for 1.5 h at 80 V. The gel was stained with GelRed^TM^ nucleic acid dye, visualized and photographed on the Bio-Rad Trans illuminator IEC 1010 (Bio-Rad, Hercules, CA).

### BamHI digestion

pUC18 contains a single restriction site (G/GATCC) which BamHI could recognize and hydrolyze the phosphodiester bond between the adjacent guanine sites. The restriction enzyme digestion of pUC18 DNA with BamHI can lead to the conversion of supercoiled form I and singly-nicked form II DNA to linear form III DNA. In this experiment, a set of drug-DNA mixtures obtained from the experiment described above was then subjected to BamHI (20 units/μL) digestion. To the solution of drug–DNA mixtures was added 2 μL of 10× digestion buffer (Cutsmart) followed by the addition of 0.1 μL BamHI (2 units). The mixtures were incubated in water bath at 37°C for 2 h. The gel was stained with GelRed^TM^ nucleic acid dye, visualized and photographed on the Bio-Rad Trans illuminator IEC 1010 (Bio-Rad, Hercules, CA).

### Maximum tolerated dose (MTD) determination

Three weeks-old male DBA/2 mice purchased from Beijing Vital River Laboratory Animal Technology Co. Ltd. were used for MTD study. Determination of MTD was performed as previously described [22]. Briefly, the animals received 5a (15, 30 and 60 mg/kg) and oxaliplatin (6, 10, 14 mg/kg) via intravenous injection on day 1, day 5 and day 9. Physiological observations (body weight and food consumption) were recorded. The maximum tolerated dose is defined as the highest tolerated dose (mean weight loss < 15%) and not cause major (life-threatening) toxicity (< 15% toxic deaths) during the study.

### Anti-leukemic activity *in vivo*

The same male DBA/2 mice were used for *in vivo* efficacy study. Mice were kept in a pathogen-free environment under a condition of constant photoperiod. Experiments were carried out at the Institute of Biomedical Engineering of the Chinese Academy of Medical Sciences (Tianjin, China) and the experimental procedure was approved by the Ethics Committee and Animal Care Committee of the Institute. Six mice for treatment groups and ten mice for control group were used for this *in vivo* study. L1210 murine leukemia cells (1 × 10^6^) were injected intraperitoneally into DBA/2 mice on day 0. Both 5a and oxalilplatin dissolved in deionized water under ultrasound for 15 min just before use. The drug solutions were administered intraperitoneally at concentrations of 11 mg/kg for 5a (equal molar to that of oxaliplatin, 18% MTD) and 7 mg/kg for oxaliplatin (70% MTD) on days 1, 5, and 9. Control group of ten mice each received injections of sterile saline. Animals were observed daily for clinical signs of toxicity and body weight was measured throughout the study. Survival times of experimental mice were recorded to evaluate the therapeutic efficacy of 5a and oxalilplatin since the day after tumor cell injection and compared to that of control animals over a period of 7 weeks.

### Anticancer activity against HT29 cells *in vivo*

Female BALB/c nude mice were inoculated by subcutaneous injection of HT29 human colon cancer cells (5 × 10^6^). For the treatment groups, drug dosage was adjusted for average body weight for each group. Injection volume was 10 mL/kg. Each treatment group contains eight mice and control animals used ten mice and received injections of sterile saline. Treatments were initiated when the tumor reached a volume of 100-300 mm^3^ in all mice. Animals received multiple treatments intravenously on days 0, 7, 14 and 21. Oxaliplatin and 5a were administered at the dose of 7 mg/kg (100% of MTD) and 25 mg/kg (70% of MTD) respectively. Saline (5% v/v) was administered to the vehicle control group. Tumor volume was assessed regularly by measuring length (l) and width (w) with caliper and calculated using the formula V = lw^2^/2. Body weight was recorded and used as a parameter of systemic toxicity. All mice were sacrificed on the 46th day of the experiment, and the tumor weight was measured.

### Statistical analysis

All the results are presented as the mean ± standard deviation (SD) and the statistical parameters used in the experiments were calculated with GraphPad Prism (version 6.02; GraphPad Software, La Jolla, CA, USA). Significant changes were assessed by unpaired Student *t* test (two-tailed). P values of <0.05 were considered statistically significant.

## SUPPLEMENTARY FIGURES



## Abbreviations

GLUT1: glucose transporter 1
SCC: squamous cell carcinoma
PET: positron emission tomography
DACH: diaminocyclohexane
GSH: glutathione
TGI: tumor growth inhibition
ICP-MS: inductively coupled plasma mass spectrometry
5′-GMP: 5′-Guanosine Monophosphate
ATCC: American Type Culture Collection
MTD: Maximum Tolerated Dose
qRT-PCR: Real-Time Quantitative Reverse Transcription PCR
PVDF: polyvinylidene difluoride
