# Supplementary material for: GLUT1-mediated selective tumor targeting with fluorine containing platinum(II) glycoconjugates
Source: Oncotarget. 2017 Apr 13;8(24):39476–96. doi: 10.18632/oncotarget.17073 (PMC5503626; doi:10.18632/oncotarget.17073)
Supplement: Supplementary file 1 [file oncotarget-08-39476-s001.pdf]

GLUT1-mediated selective tumor targeting with fluorine containing platinum(II) glycoconjugates

SUPPLEMENTARY MATERIALS

SUPPLEMENTARY FIGURES AND TABLES

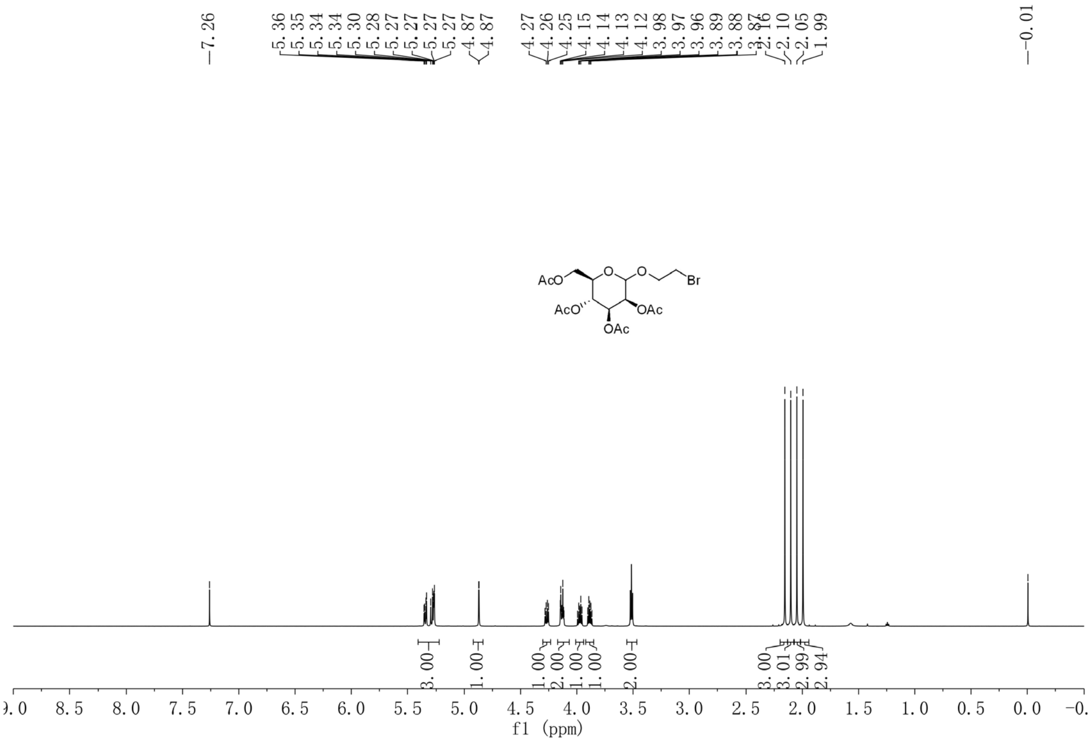

Supplementary Figure 1: ^{1}H-NMR of compound 1b.

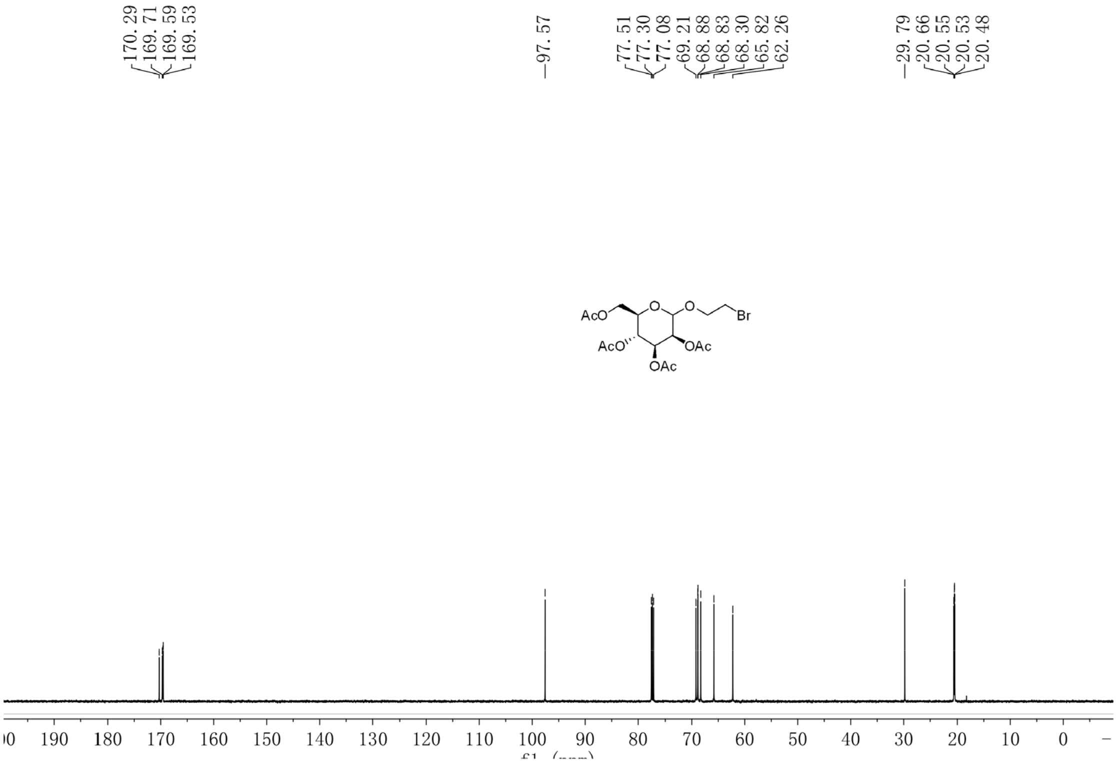

Supplementary Figure 2: ^{13}C-NMR of compound 1b.

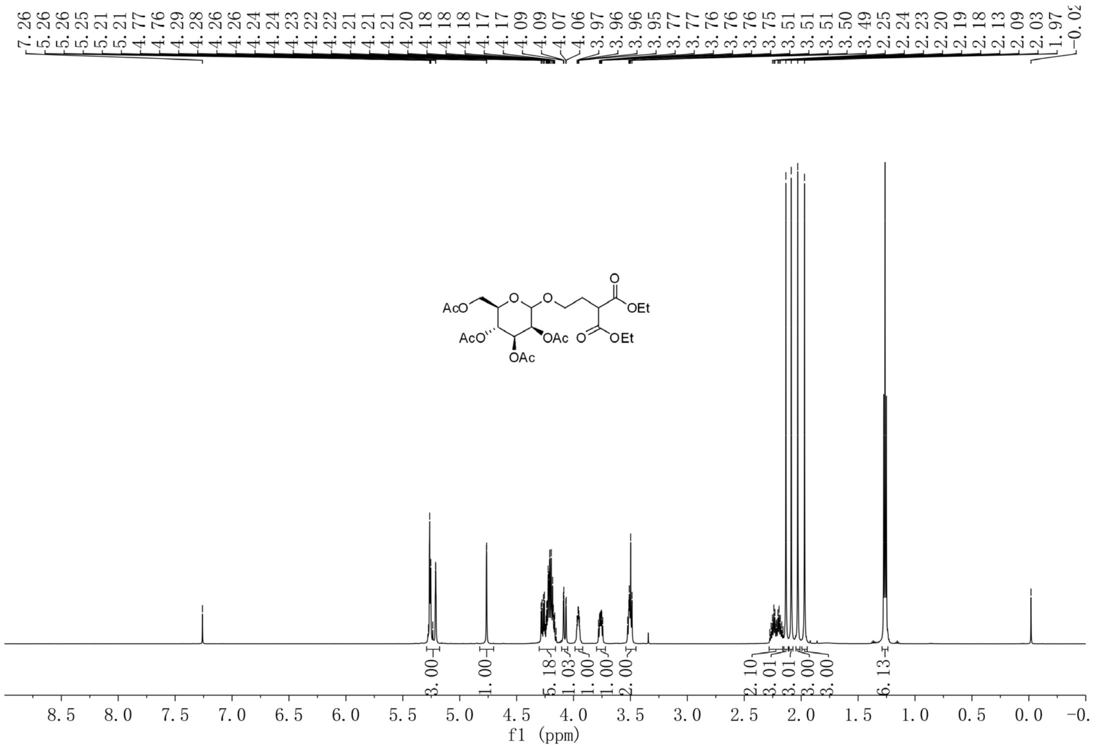

Supplementary Figure 3: ^{1}H-NMR of compound 2b.

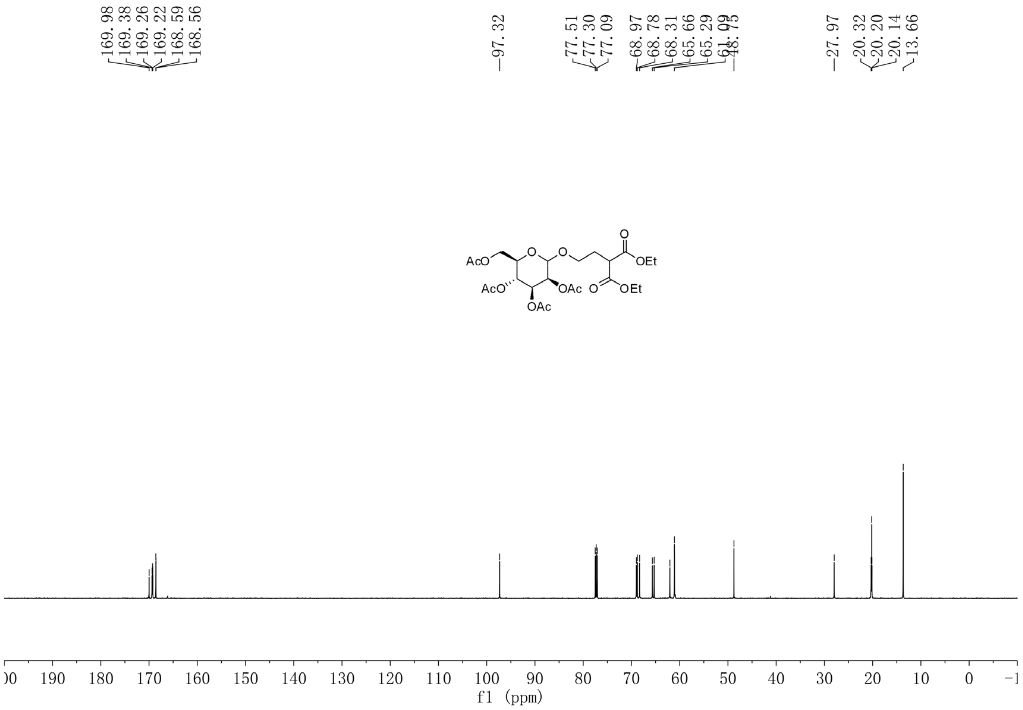

Supplementary Figure 4: ^{13}**C-NMR of compound 2b.**

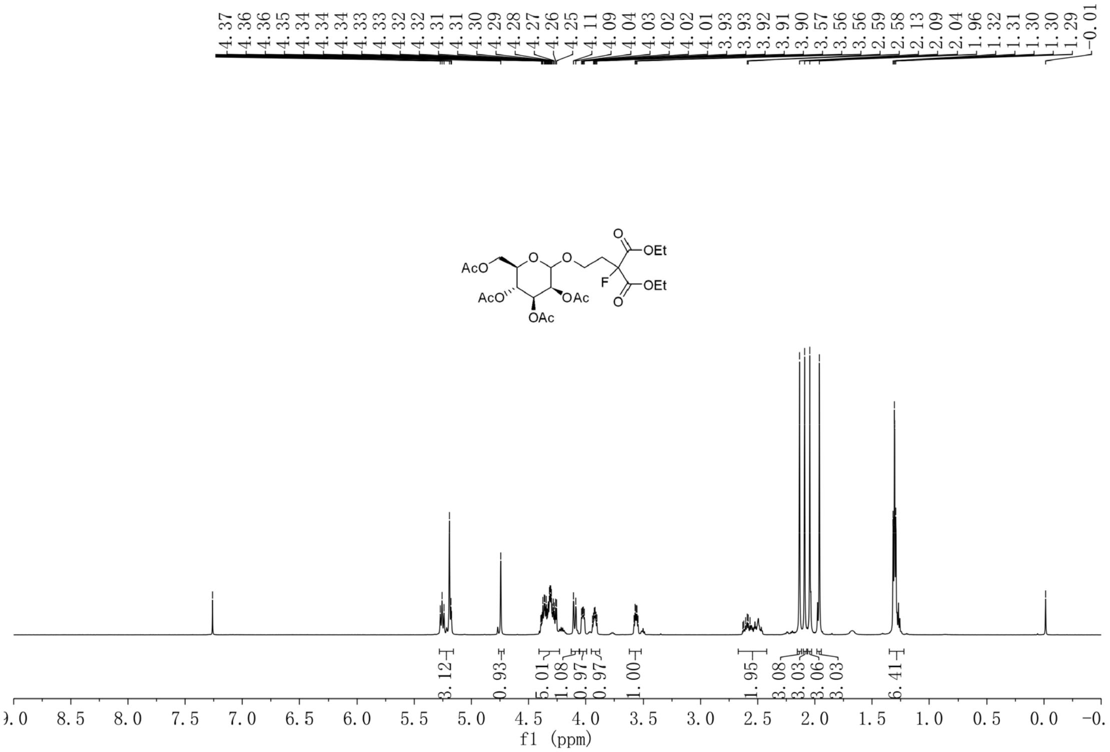

Supplementary Figure 5: ^{1}H-NMR of compound 3b.

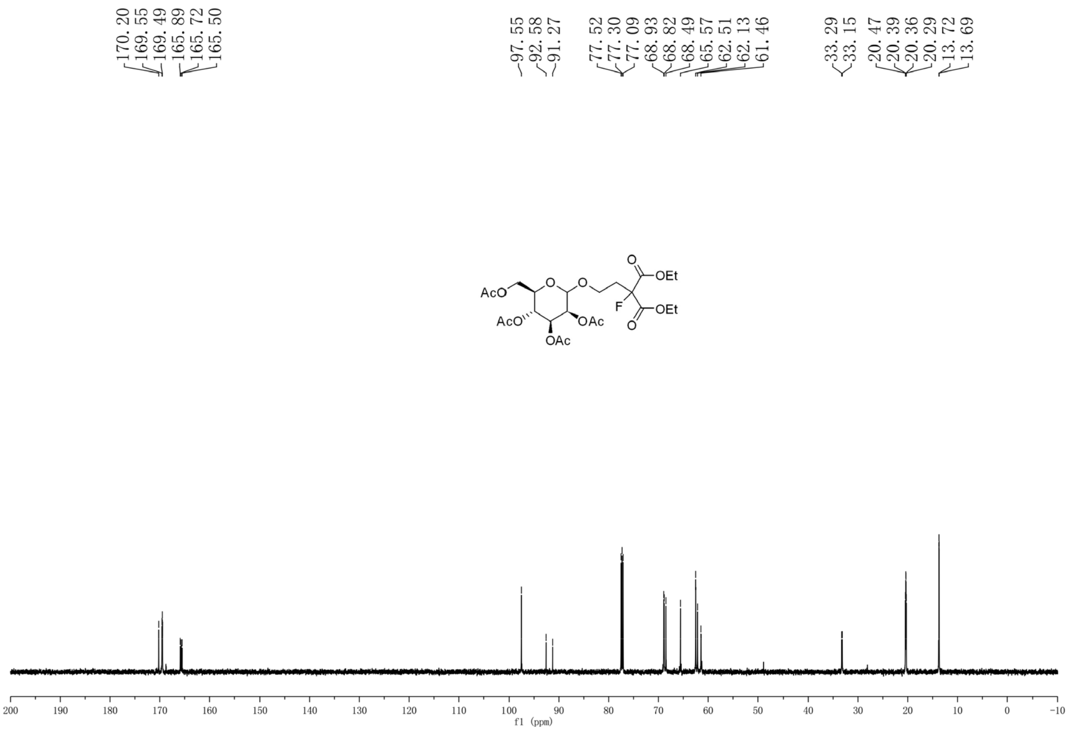

Supplementary Figure 6: ^{13}C-NMR of compound 3b.

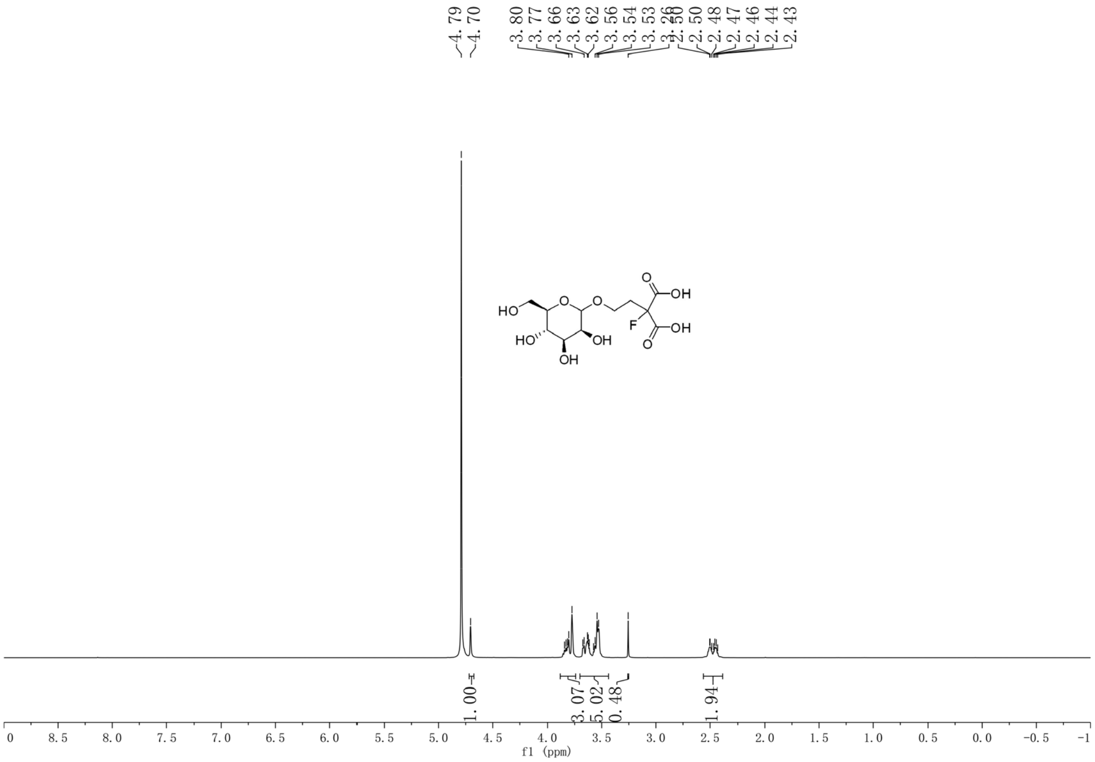

Supplementary Figure 7: ^{1}H-NMR of compound 4b.

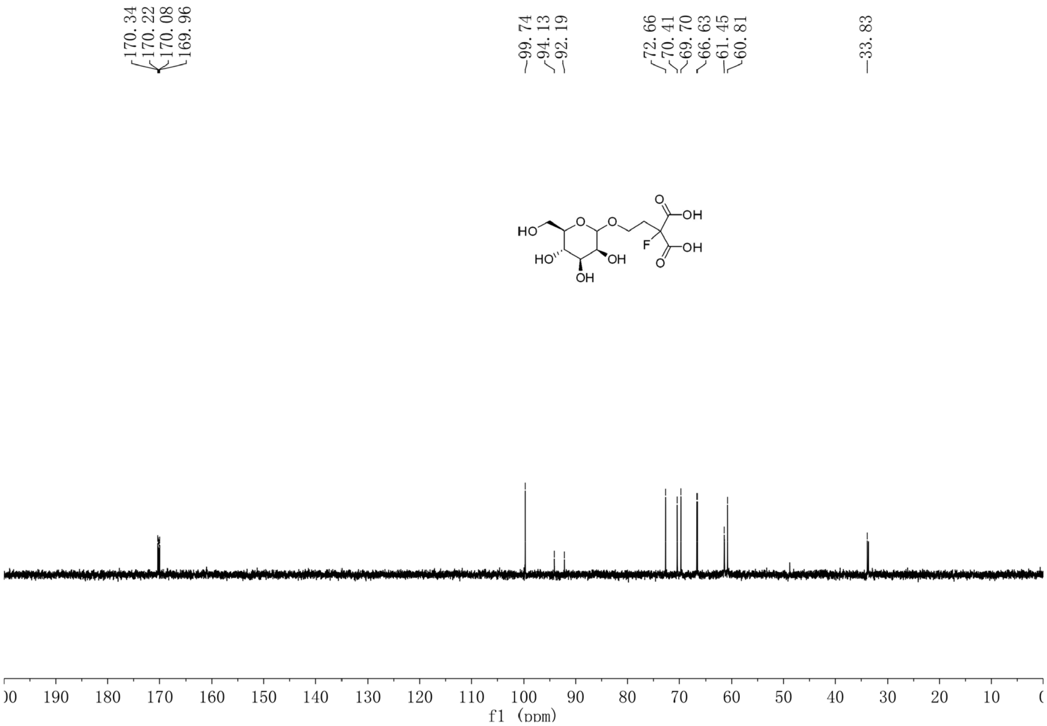

Supplementary Figure 8: ^{13}C-NMR of compound 4b.

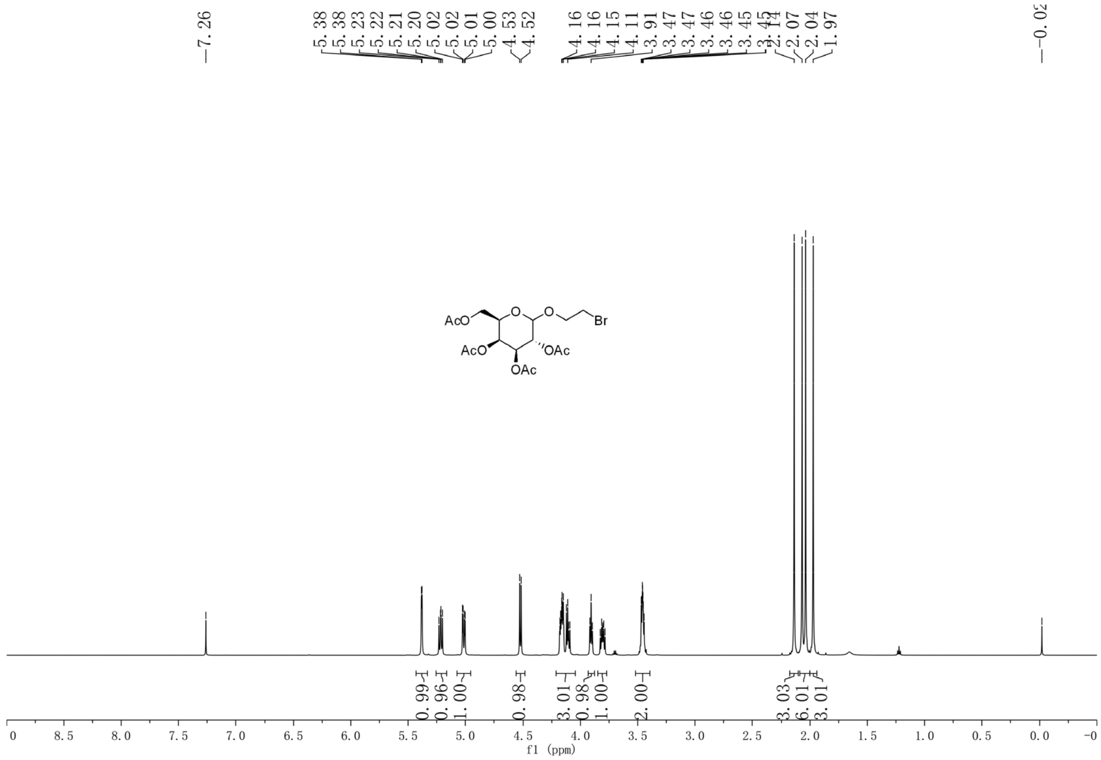

Supplementary Figure 9: ^{1}H-NMR of compound 1c.

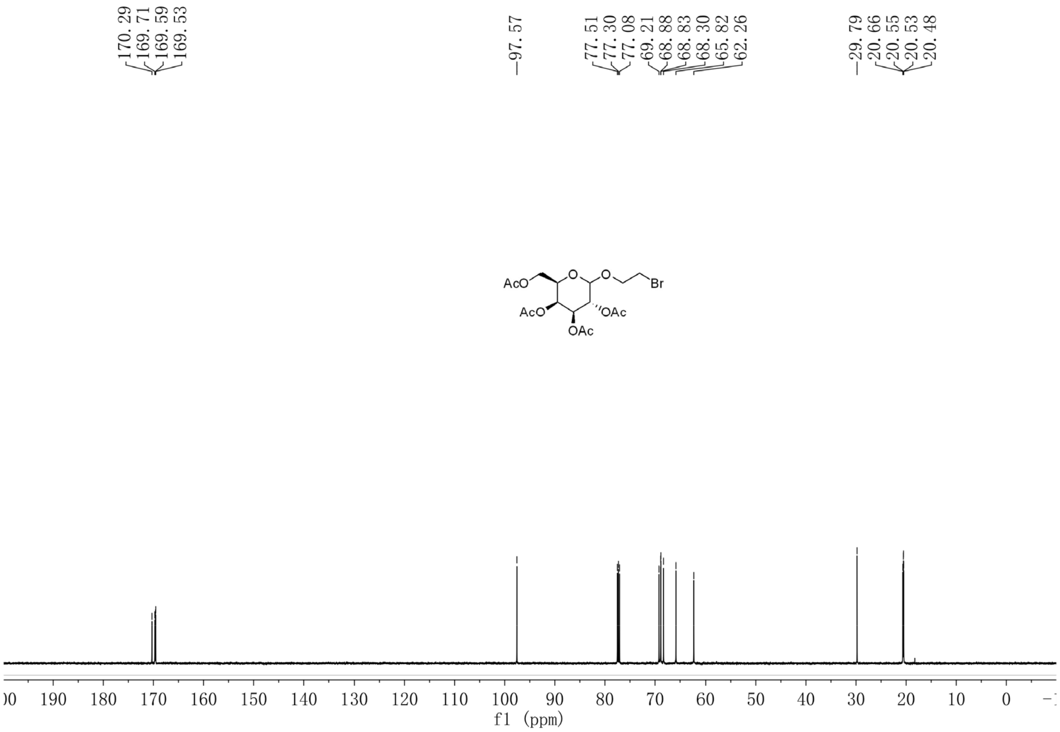

Supplementary Figure 10: ^{13}C-NMR of compound 1c.

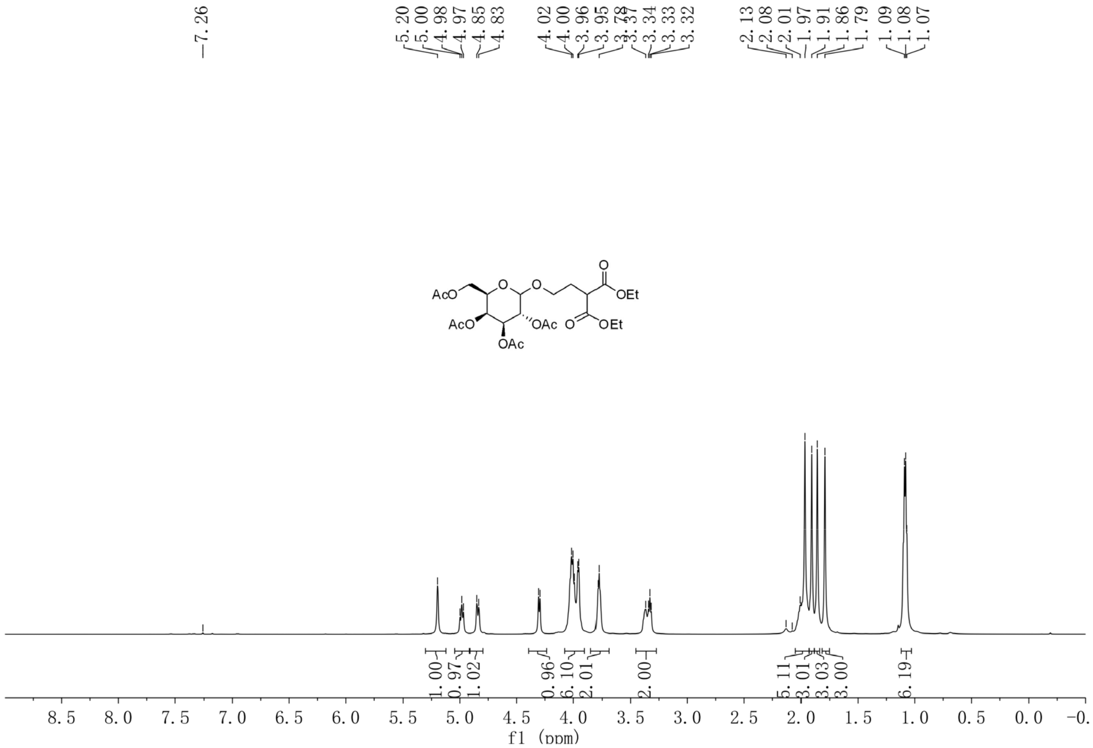

Supplementary Figure 11: ^{1}H-NMR of compound 2c.

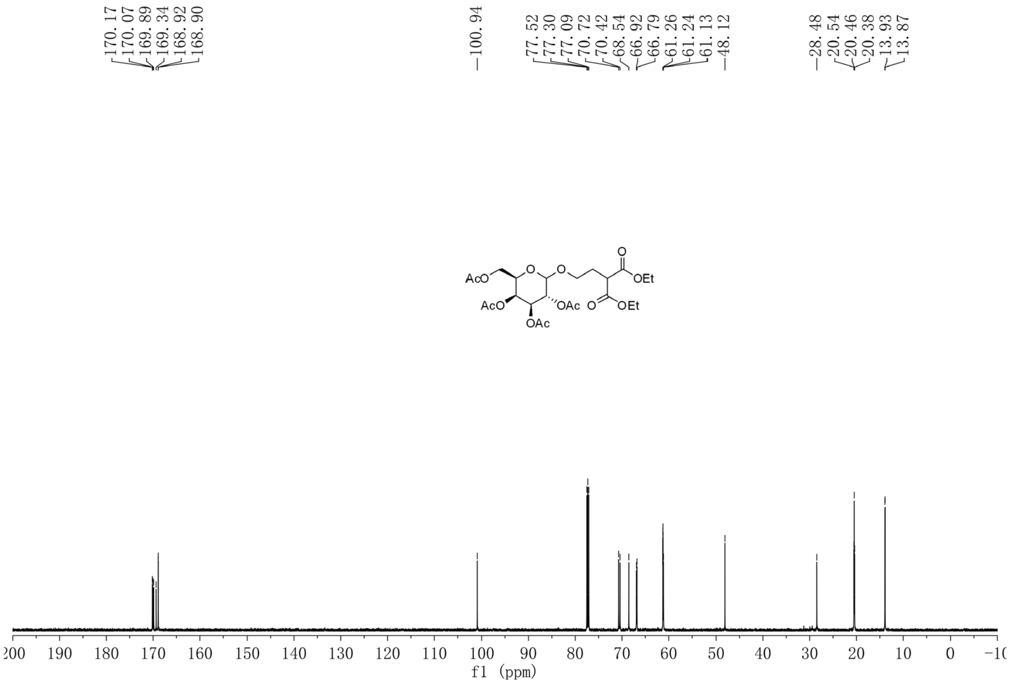

Supplementary Figure 12: ^{13}C-NMR of compound 2c.

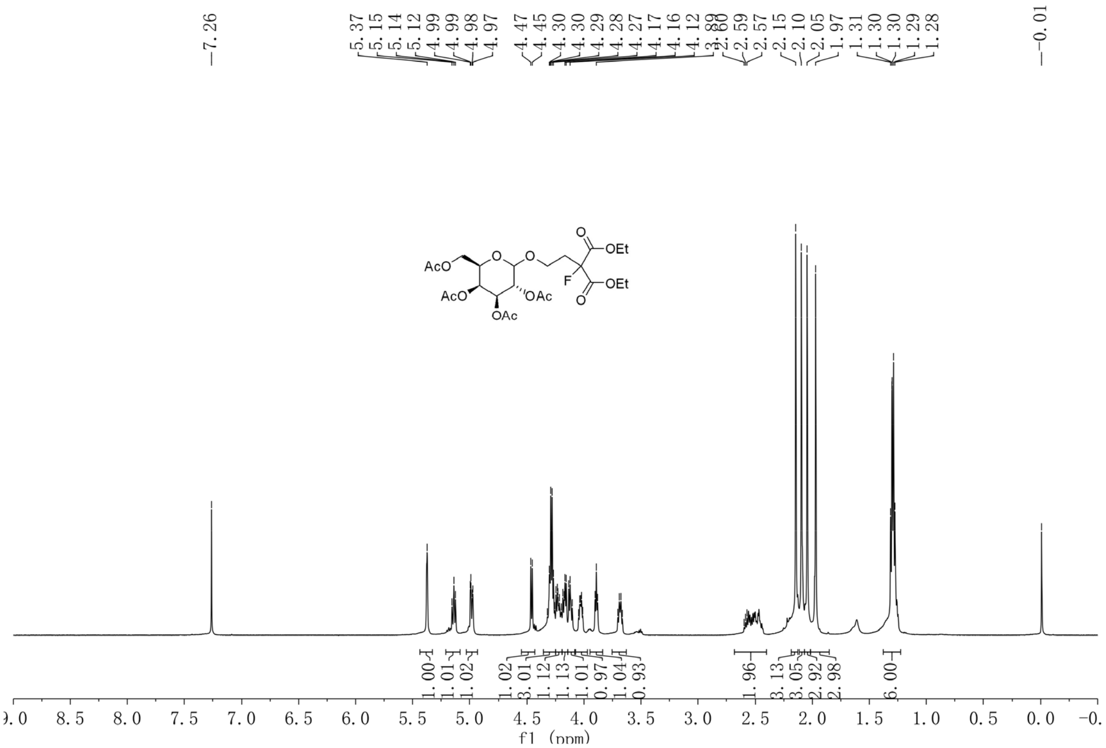

Supplementary Figure 13: ^{1}H-NMR of compound 3c.

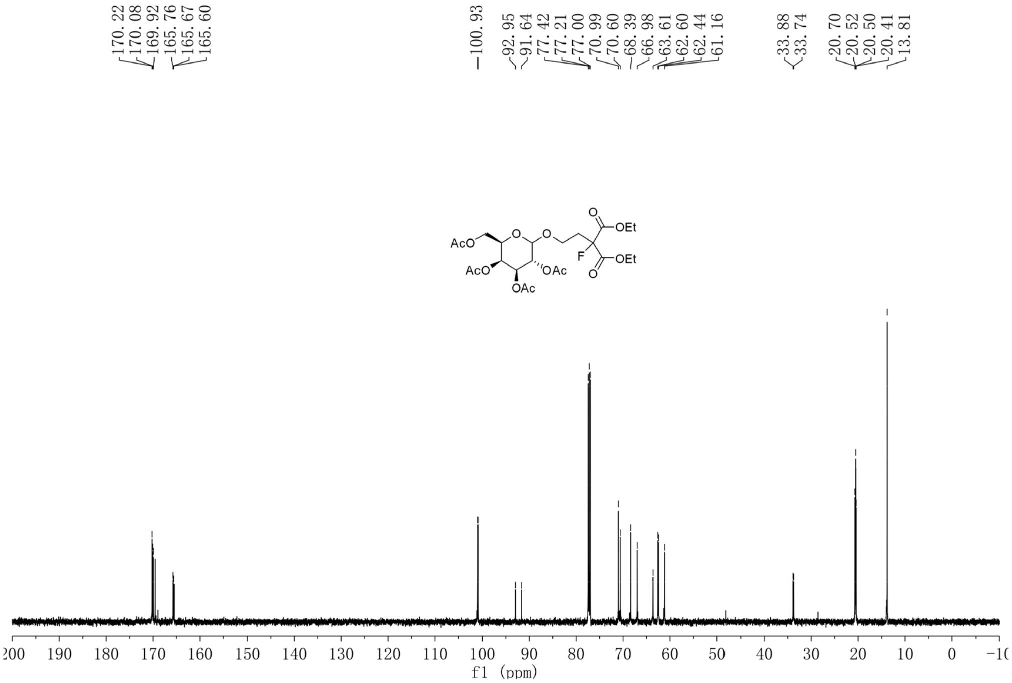

Supplementary Figure 14: ^{13}C-NMR of compound 3c.

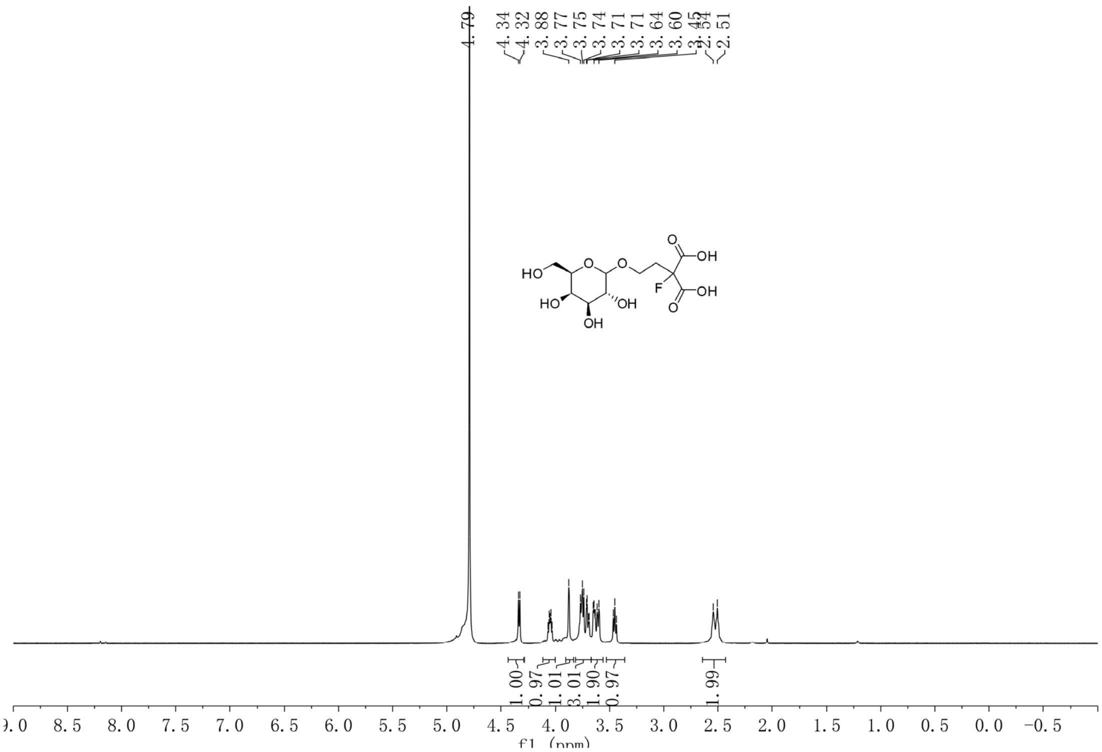

Supplementary Figure 15: ^{1}H-NMR of compound 4c.

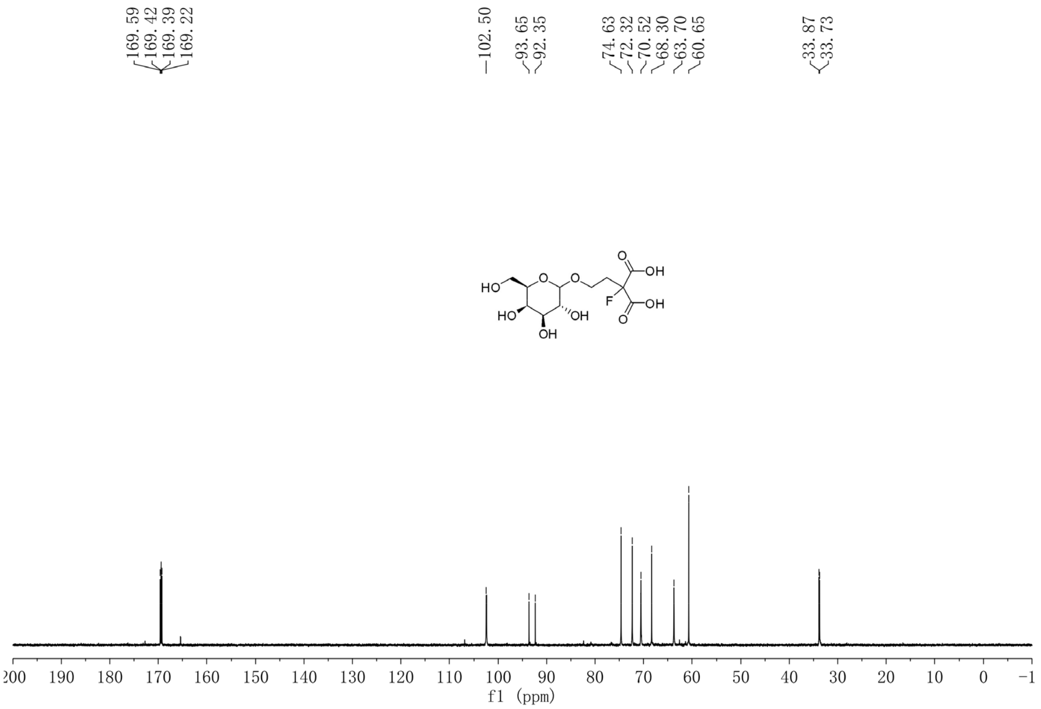

Supplementary Figure 16: ^{13}C-NMR of compound 4c.

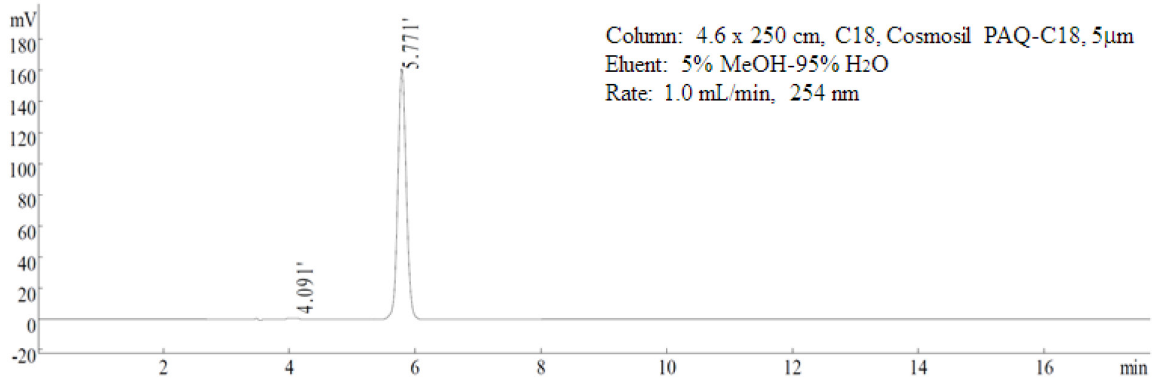

No.	RT (min)	Concentration(%)	Area	Height
1	4.091	2.91	45455	1522
2	5.771	97.09	1516636	161993

Supplementary Figure 17: Purity confirmation of 5a by HPLC.

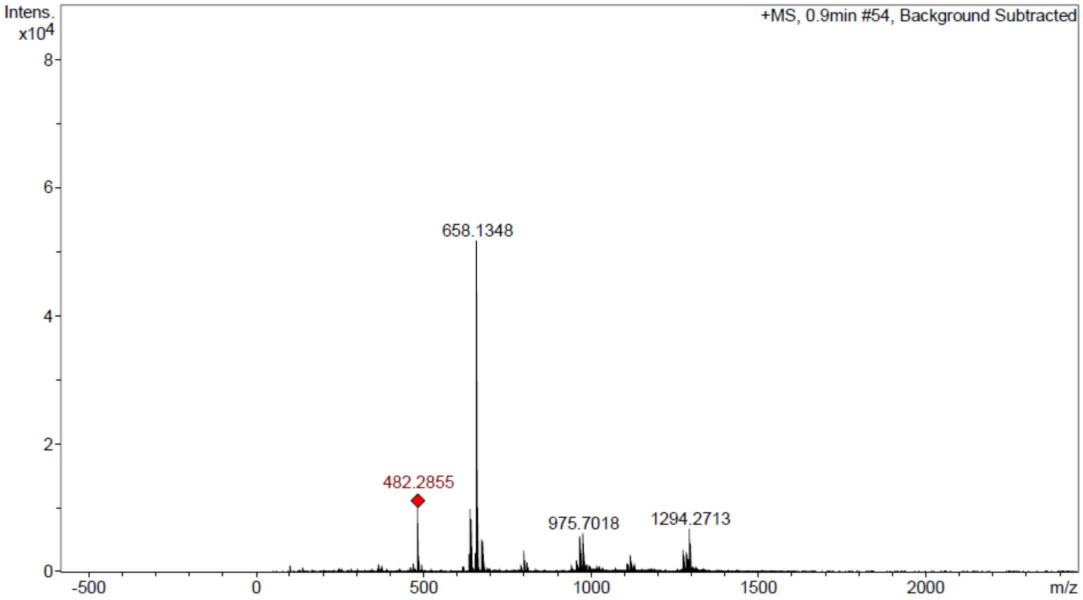

Supplementary Figure 18: Mass spectrum of compound 5a.

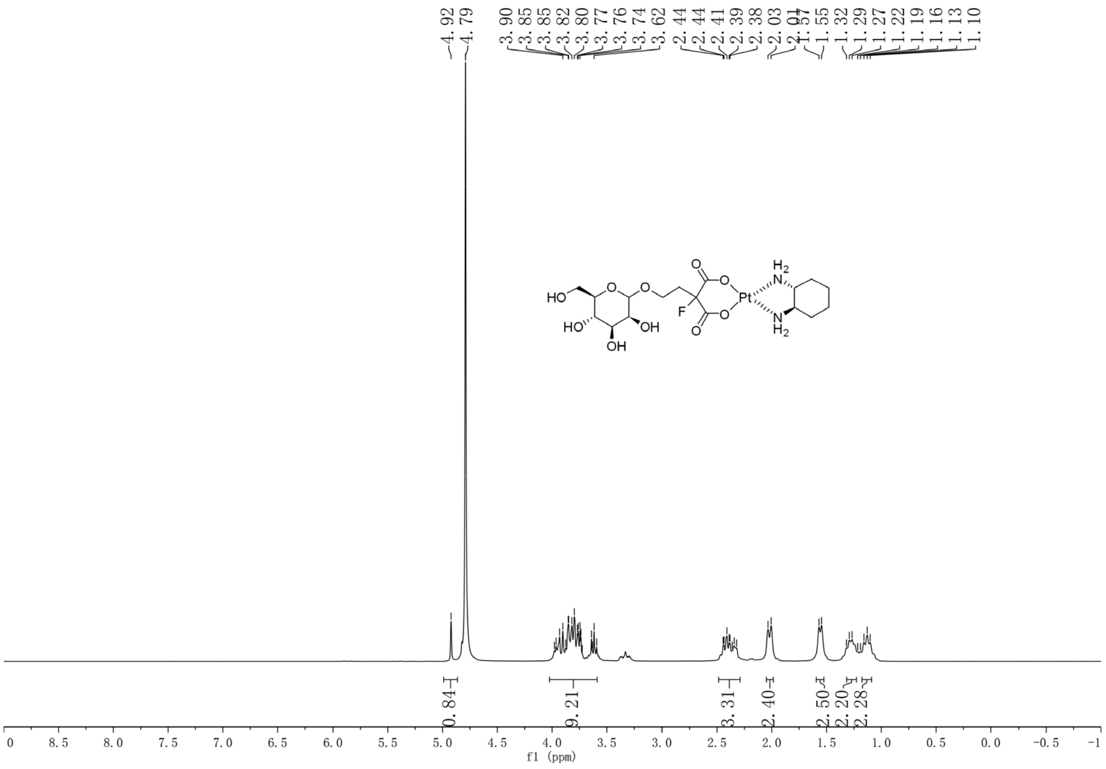

Supplementary Figure 19: ^{1}H-NMR of compound 5b.

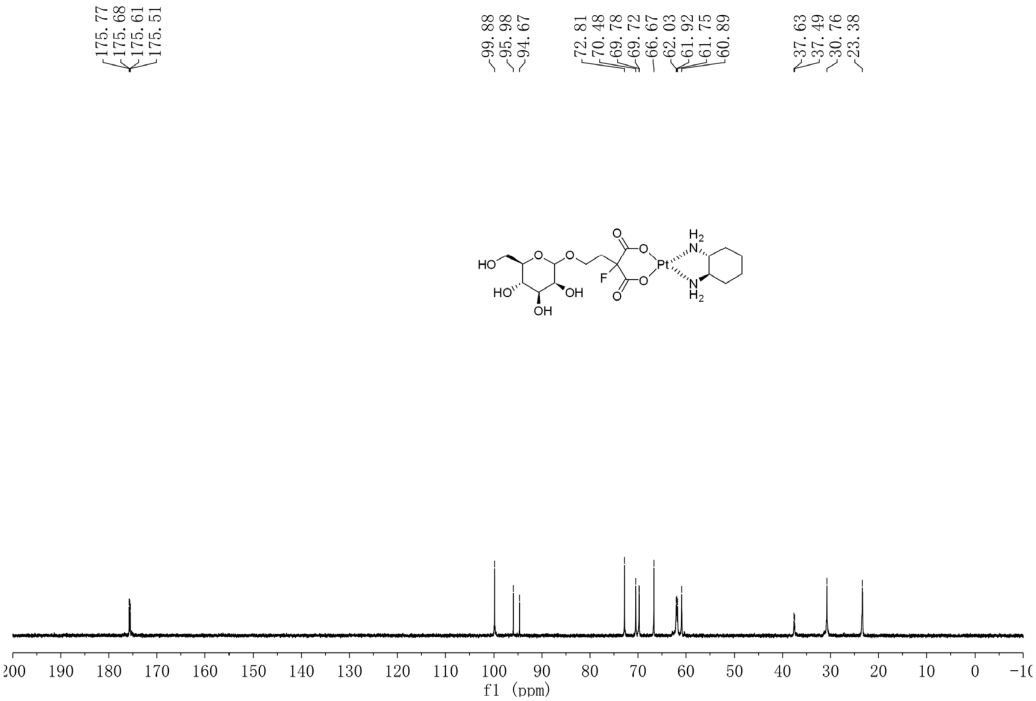

Supplementary Figure 20: ^{13}C-NMR of compound 5b.

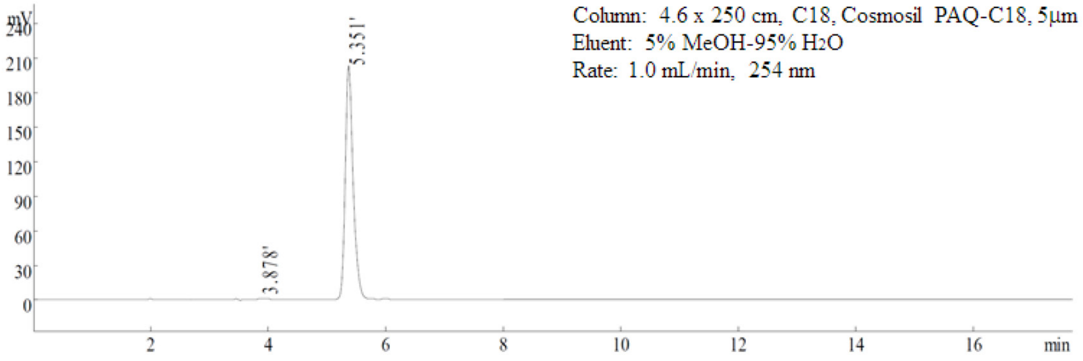

No.	RT (min)	Concentration(%)	Area	Height
1	3.878	1.547	29820	1530
2	5.351	98.46	1897343	203958

Supplementary Figure 21: Purity confirmation of 5b by HPLC.

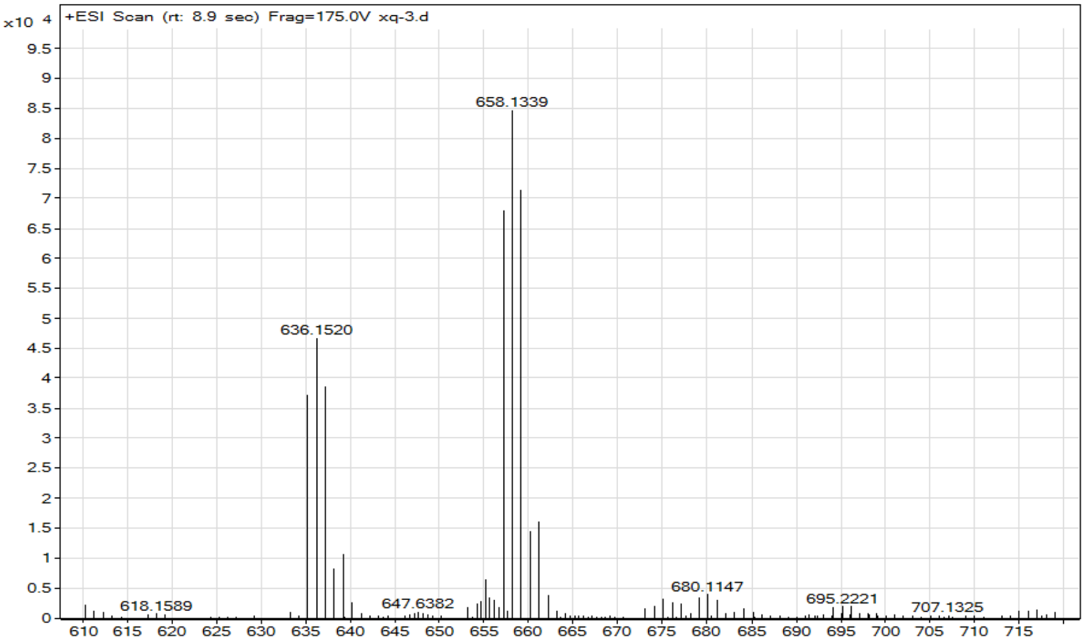

Supplementary Figure 22: Mass spectrum of compound 5b.

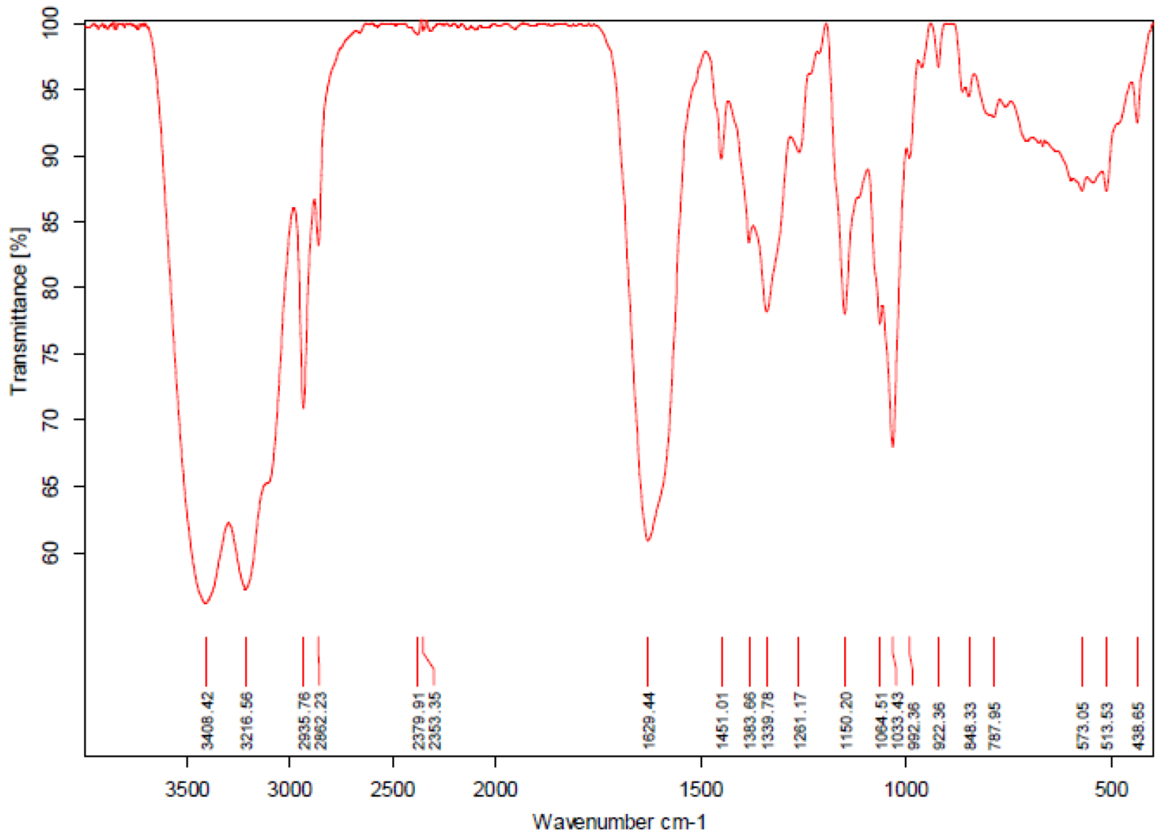

Supplementary Figure 23: IR spectrum of compound 5b.

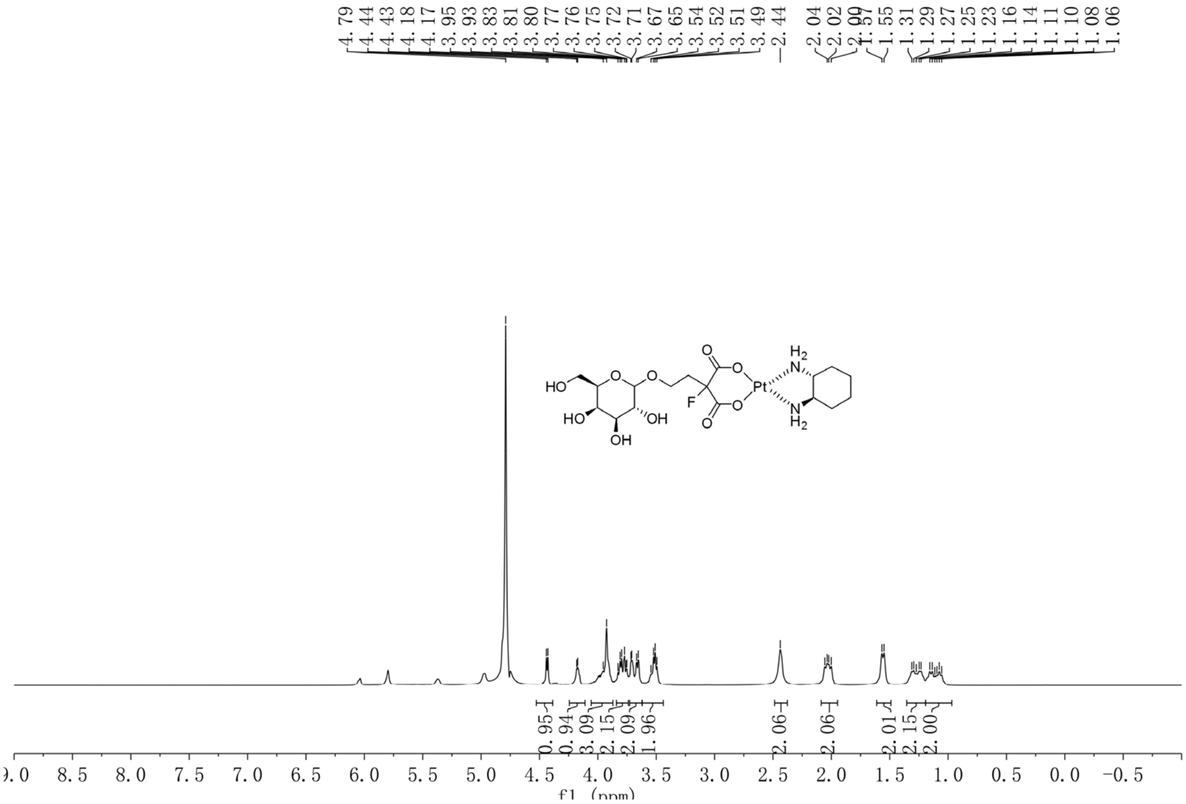

Supplementary Figure 24: ^{1}H-NMR of compound 5c.

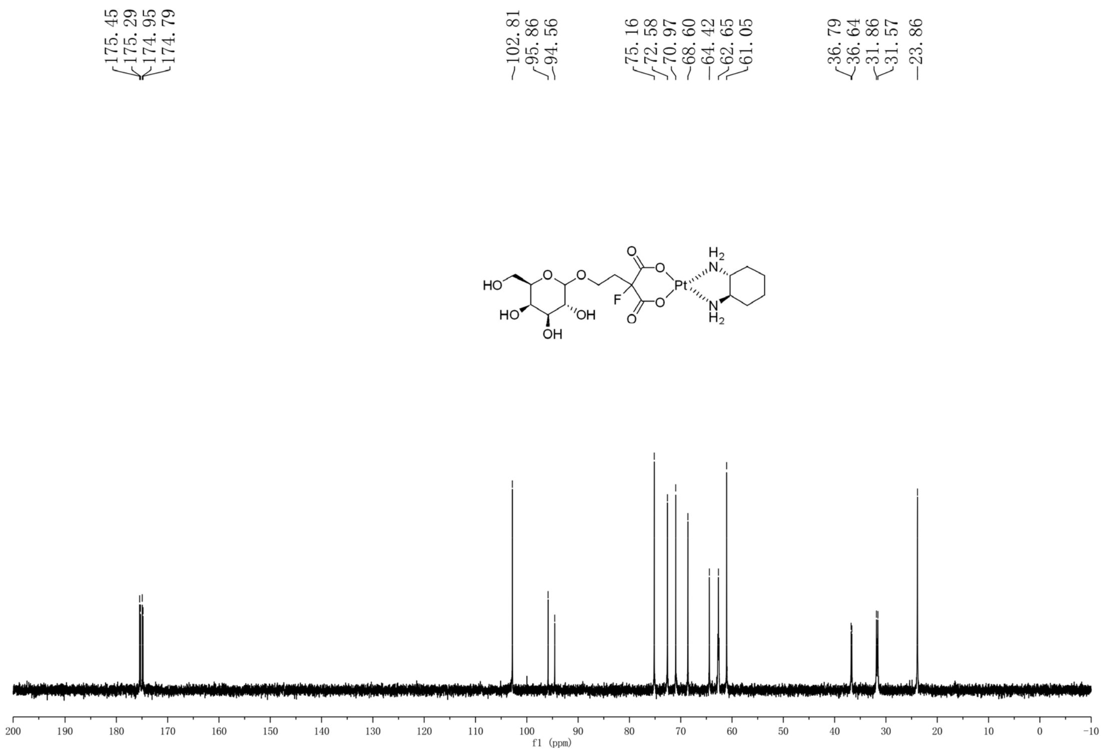

Supplementary Figure 25: ^{13}C-NMR of compound 5c.

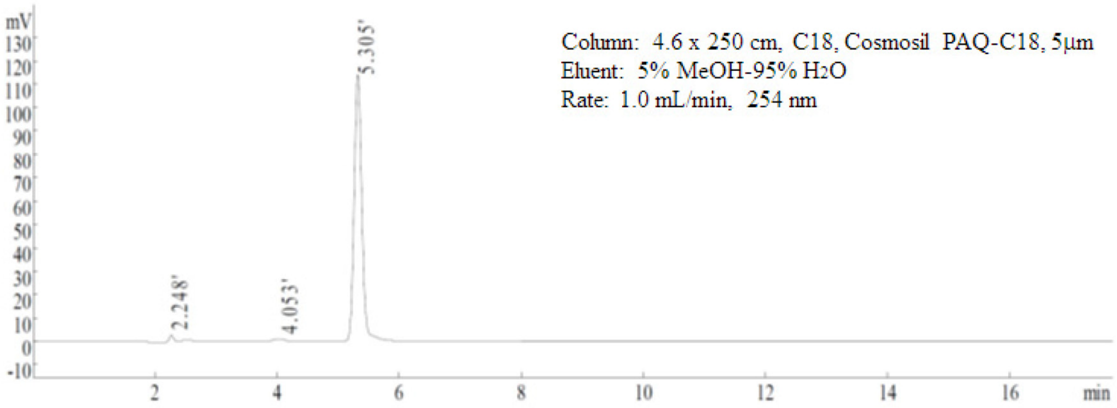

No.	RT (min)	Concentration(%)	Area	Height
1	2.248	1.594	16525	3292
2	4.053	1.439	14922	1012
3	5.305	96.96	1005108	114608

Supplementary Figure 26: Purity confirmation of 5c by HPLC.

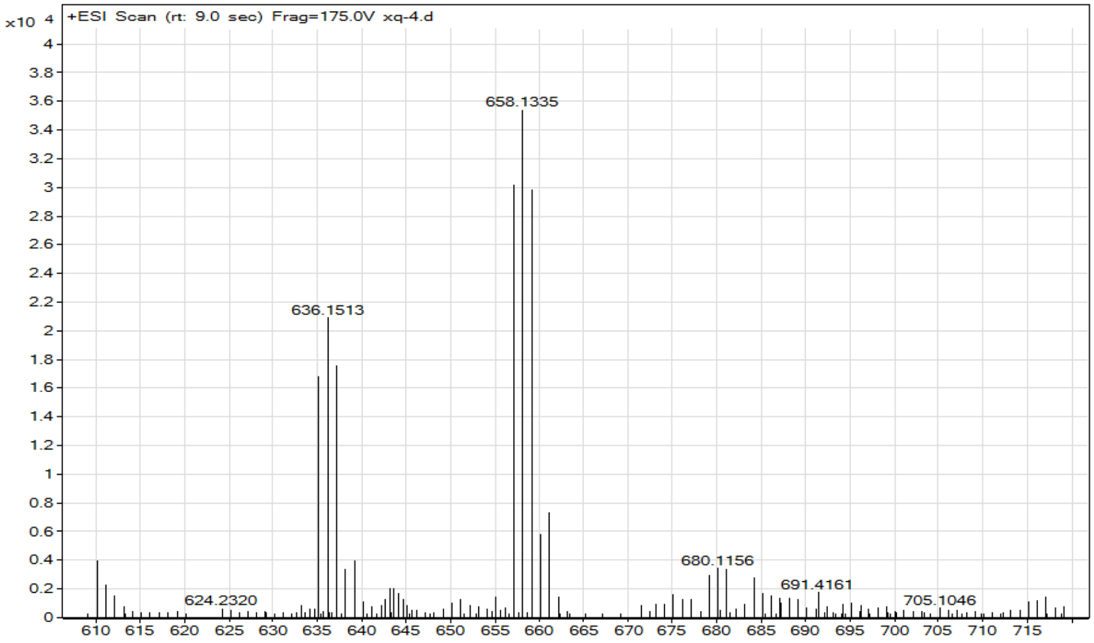

Supplementary Figure 27: Mass spectrum of compound 5c.

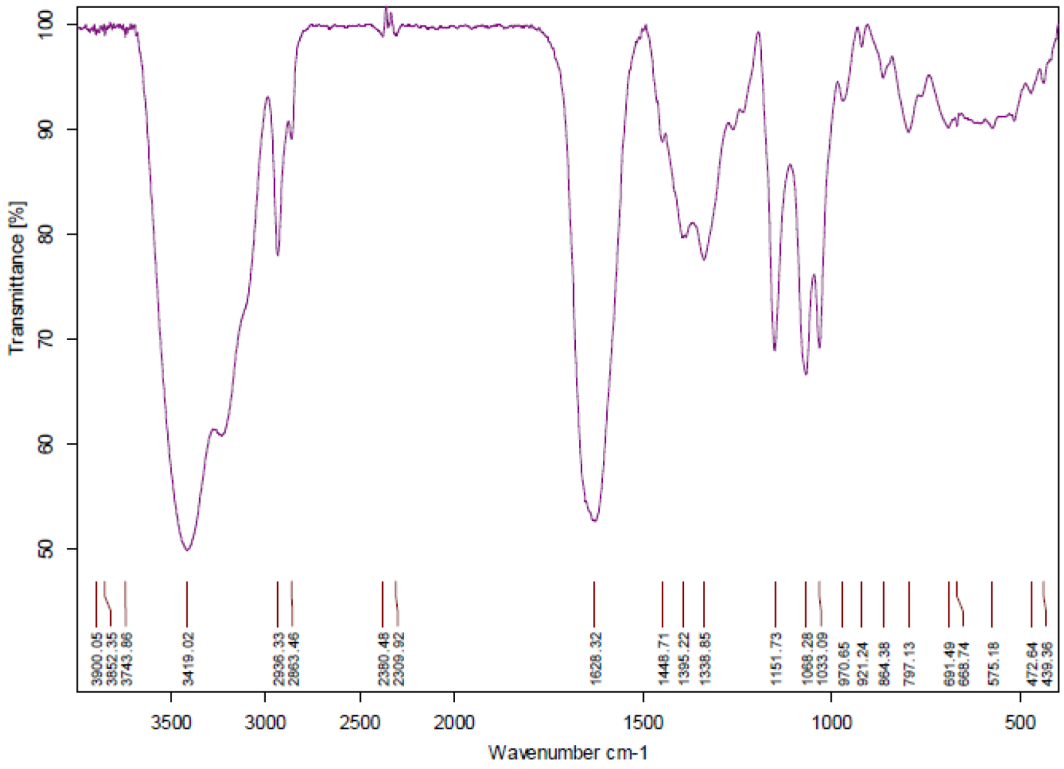

Supplementary Figure 28: IR spectrum of compound 5c.

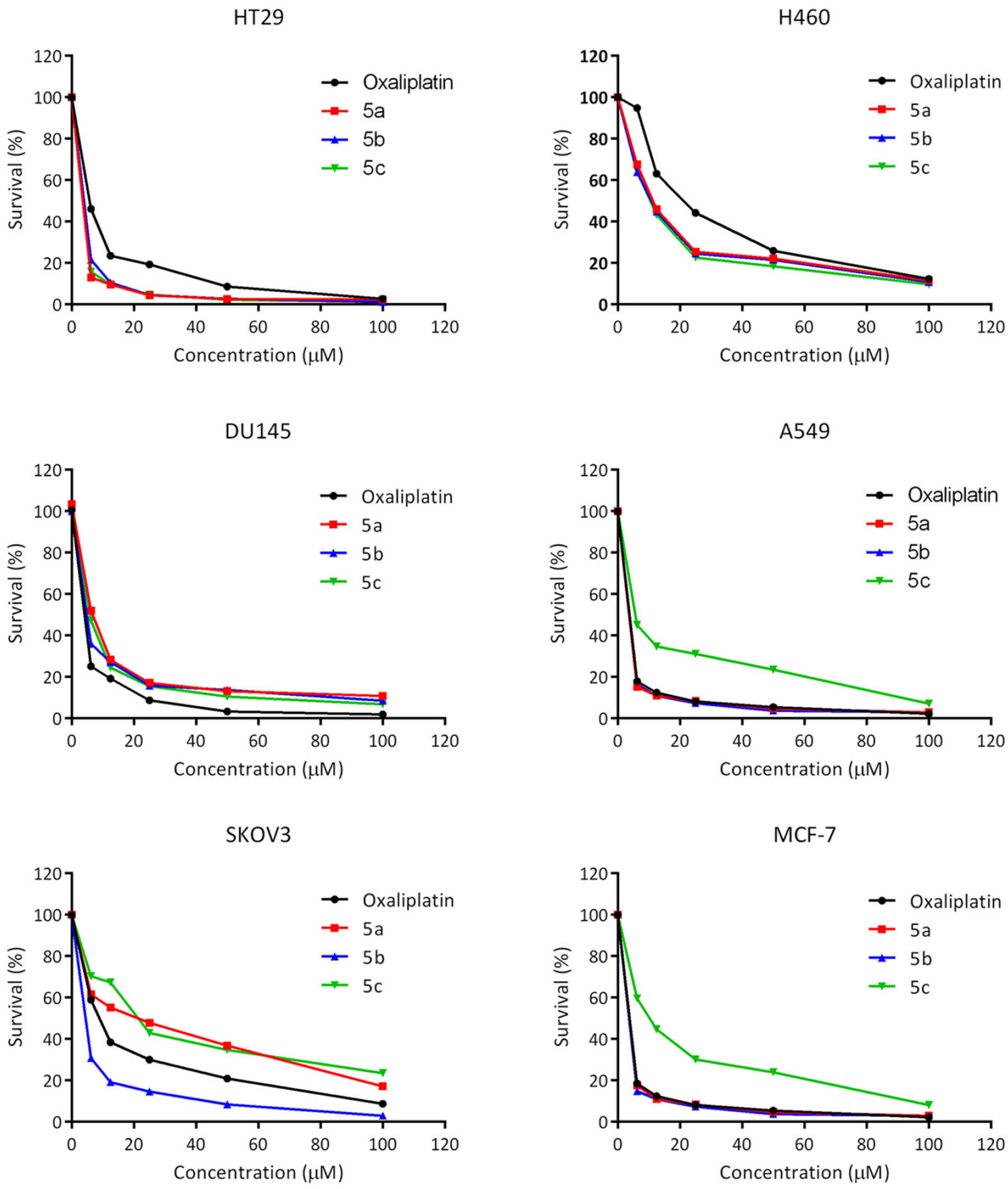

Supplementary Figure 29: Cytotoxicity assays (MTS assays). concentration response curves of six cancer cell lines (HT29, H460, DU145, A549, SKOV3, MCF-7) treated with sugar conjugated platinum complexes 5a, 5b, 5c and oxaliplatin.

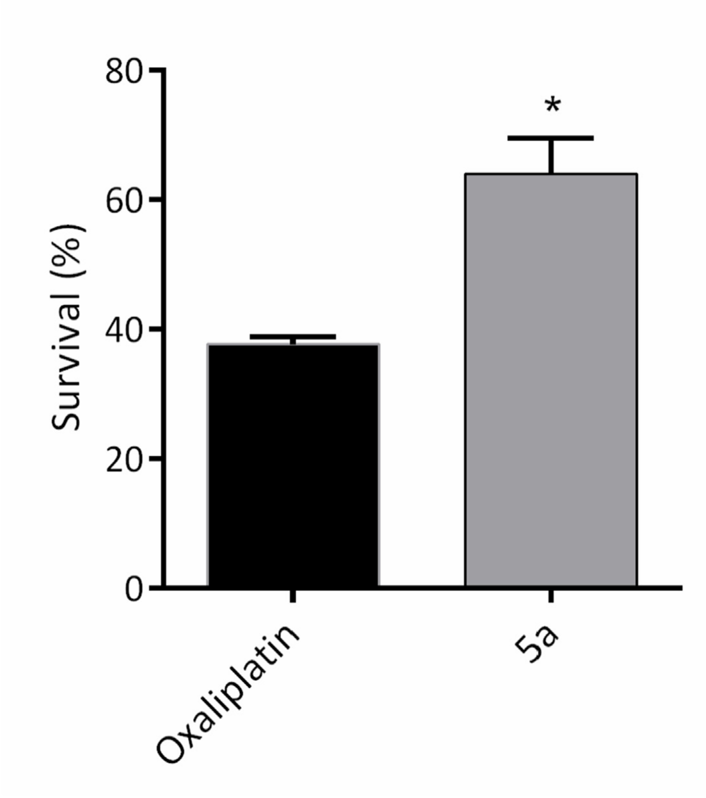

Supplementary Figure 30: Cytotoxicity assays on EBAS-2B-CM. suvival rates of normal epithelial cells after 72 h treatment with sugar conjugated platinum complexes 5a and oxaliplatin (6.25µM each) (* $p < 0.05$ compared to oxaliplatin group).
